# Biological Activities of Novel Oleanolic Acid Derivatives from Bioconversion and Semi-Synthesis

**DOI:** 10.3390/molecules29133091

**Published:** 2024-06-28

**Authors:** Nahla Triaa, Mansour Znati, Hichem Ben Jannet, Jalloul Bouajila

**Affiliations:** 1 Medicinal Chemistry and Natural Products Team, Laboratory of Heterocyclic Chemistry, Natural Products and Reactivity (LR11ES39), Faculty of Science of Monastir, University of Monastir, Avenue of Environment, Monastir 5019, Tunisia; triaanahla19@gmail.com (N.T.); znatimansour@yahoo.fr (M.Z.); 2 Laboratoire de Génie Chimique, Université Paul Sabatier, CNRS, INPT, UPS, 31062 Toulouse, France

**Keywords:** oleanolic acid, triterpenoids, medicinal chemistry, biotransformation, biological activities, anticancer activity, pharmaceutical properties

## Abstract

Oleanolic acid (OA) is a vegetable chemical that is present naturally in a number of edible and medicinal botanicals. It has been extensively studied by medicinal chemists and scientific researchers due to its biological activity against a wide range of diseases. A significant number of researchers have synthesized a variety of analogues of OA by modifying its structure with the intention of creating more potent biological agents and improving its pharmaceutical properties. In recent years, chemical and enzymatic techniques have been employed extensively to investigate and modify the chemical structure of OA. This review presents recent advancements in medical chemistry for the structural modification of OA, with a special focus on the biotransformation, semi-synthesis and relationship between the modified structures and their biopharmaceutical properties.

## 1. Introduction

Plants have been a vital source of nourishment and medicinal substances for mankind for a long time [1]. This source is characterized by a diversity of molecules with a variety of bioactive properties [2]. Therefore, natural products have several notable advantages, including biodegradability, wide availability from diverse sources, and low susceptibility to drug resistance [3]. Nature has been used since ancient times to combat various illnesses [4]. Per the World Health Organization (WHO), 80% of developing countries’ populations rely on the use of effective traditional medical practices as their principal form of health care [5]. Chemists worldwide have shown great interest in natural source products due to their potential to provide new chemical varieties for drug discovery [6,7]. Almost one half of the new medicines launched in the last three out decades are either naturally occurring products or their derivatives. 

According to the WHO, many countries, including Germany (77%), France (49%), Belgium (31%), Australia (48%), and Canada (70%), have adopted traditional herbal treatment systems [8]. Additionally, traditional Chinese herbal medicine has been used to treat COVID-19 [9]. Triterpenoids are a valuable benchmark for drug discovery programs because of their wide diversity of activities. To date, more than 20,000 triterpenoids have been discovered [10,11]. Triterpenoid compounds are a type of secondary metabolite [12] with a diverse range of biopharmaceutical activities, among them anti-inflammatory [13], antiviral activity against HIV [14], antidiabetic, neuropharmacological [15] and antihyperuricemic properties [16]. Pentacyclic triterpenoids, such as lupane, oleanane, and ursane [17], exhibit bioactivity [18]. Among these, Oleanolic acid (OA) has received more attention from researchers due to its abundance in medicinal herbs and foods [19]. Discovered in the 1970s, this molecule is chemically known as 3β-hydroxyolean-12-en-28-oic acid [20,21] and is also known as angelic acid, caryophellin and oleanol [22]. It is derived from the oleanane family of pentacyclic triterpenoids [23,24] and is extracted from over 2000 plants [25], including numerous food and medicinal herbs [26]. For example, the following plants have been identified as containing this compound: *Corni Fructus* [27], *Salvia* [28], *Olea europea* [29], *Pistacia lentiscus* [30], *Apples* [31], *Viscum album* L. [32], *Aralia elata* [33], *Couepia polyandra*, *Perilla frutescens*, and *Glechoma hederaceae* [34]. Naturally, OA can be present as a free acid. It is also found as an aglycone precursor of the triterpenoid saponins, where it can be associated with sugar or sugar chains [20]. For instance, numerous oleanolic acid saponins are derived from the *Viguiera decurrens* plant, including 15a-angeloyloxy-ent-kaur-16-en19-oic acid, oleanolic acid-3-*O*-methylb-d-glucuronopyranosiduronoate, etc. These compounds exhibit intriguing anti-cancer properties [35]. Additionally, 3-*O*-α-l-arabinosyl oleanolic acid can be isolated from *Schumacheria castaneifolia* and has interesting anticancer activity [36]. OA has been used in traditional medicines for centuries; it is an ingredient in traditional Chinese medicine (TCM) and has been clinically used for 20 years to treat hepatitis [37]. It is also widely used in India as a medicinal compound with natural properties [38]. Today, many medicines are derived from plants, underlining the importance of traditional remedies in modern medicine. The therapeutic potential of *Ocimum sanctum* L. is well documented, particularly as an anti-asthmatic and antikaphytic medicine [39]. *Azadirachta Indica* A. Juss, or neem, is a popular medicinal plant in Asia and Africa, and has been used since ancient times for a variety of purposes [40]. *Tribulus terrestris* is also used to treat urinary disorders, hyperuricaemia and impotence, as well as being a diuretic [41]. 

In a pharmacological context, the anti-apoptotic [42] and antioxidant [43] properties of oleanolic acid (OA) could well explain its various therapeutic effects. By protecting cells and reducing oxidative stress, these mechanisms can reduce hypoglycemic [44], anti-inflammatory [45], anti-cancer, anti-microbial [42] and anti-influenza [46] effects. These properties, along with its traditional medicinal use, have led researchers to consider this compound to have therapeutic potential for the prevention and control of many illnesses, including diabetes, cancer, AIDS and many other diseases [47]. Despite being widely used in various fields, the efficacy of OA has not been fully revealed as its poor solubility in water and the permeability of the cell membrane limit its use [48]. This has prompted scientific researchers to devote more attention to improving its use. Several reviews have been published on this acid, focusing on its beneficial properties and its derivatives. Yang et al. [49] analyzed recent research on semi-synthetic derivatives, with their study focusing on the advances made in understanding the biological characteristics of OA and its derivatives. However, comprehensive evaluations are lacking due to the numerous articles published each year, which presents obstacles for future research. Therefore, we have provided an update to address this issue. 

This study presents an exhaustive analysis of oleanolic acid, encompassing both biological and chemical aspects. Firstly, the enzymatic method is described, including an overview of the phenomenon and definitions of the enzymes and fungi used for bioconversion. Secondly, we offer more detail on semi-synthesis, as almost all the derivatives are semi-synthetic. A comprehensive presentation of the derivatives is provided, accompanied by detailed diagrams illustrating the chemical reactions, including the reagents and solvents utilized. Furthermore, we have addressed the biological aspect by elucidating the phenomenon of biotransformation and enzymatic reactions in general. This approach has been designed with the intention of facilitating the work of scientific researchers. In conclusion, this review can serve as a biological and organic reference for future therapeutic development.

## 2. Enzymatic Production of Oleanolic Acid Derivatives

**Figure 1:**
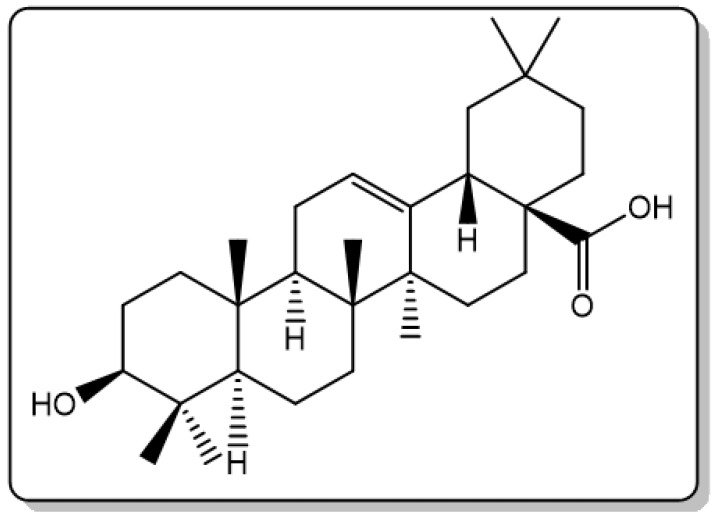
Chemical structure of oleanolic acid.

The primary objective of life is to maintain optimal health by actively combating disease, regardless of the means employed, whether simple or complex. Researchers are continuously working to discover natural molecules or synthesize compounds with intriguing biological activities. OA (Figure 1) is a pentacyclic triterpenoid that has been extensively researched and is considered highly important in nature [50]. 

Scientists have made significant efforts to improve the activity of organic compounds, whether enzymatically or chemically. Enzymatic reactions, using microorganisms, are preferred due to their simplicity, safety, and efficiency in modifying organic compounds [51,52]. Currently, there are few works on the biotransformation of OA. In fact, this review presents all derivatives of this triterpenoid acid that result from enzymatic transformations (Table 1). 

Zhang et al. [53] have described the formation of a new molecule, OA methyl ester (**1**). This molecule is characterized by the esterification of the carboxyl group located at C28. The transformation was accomplished using the bacterium *Nocardia sp. NRRL 564*. In a previous work, Choudhary et al. [50] demonstrated that the fungus *Fusarium lini* can biotransform our acid by producing two oxidative metabolites (Table 1). These compounds are distinguished by the insertion of a hydroxyl group at C2 for 2α,3β-dihydroxyolean-12-en-28-oic acid (**2**) and at C2 and C11 for 11β-trihydroxyolean-12-en-28-oic acid (**3**). Both molecules were tested for their α-glucosidase inhibition properties. The results show that the enzyme was more strongly inhibited by these two compounds, which exhibited IC_50_ values of 444 µM and 666 µM, respectively. Furthermore, Liu et al. [52] utilized two types of fungi to produce nine derivatives of OA. Six products were produced by *Alternaria longipes* through biotransformation, while *Penicillium adametzi* yielded three compounds. Four of these derivatives demonstrated greater cytotoxicity against cancerous human cell lines. Martinez et al. [54] used the fungus *Rhizomucor miehei* to hydroxylate C-1, C-7, and C-30 (**13**–**15**). In addition, Ting et al. [51] carried out a microbiological conversion of OA using *Trichothecium roseum*, resulting in the discovery of two new hydroxylated compounds, 15α-hydroxy-3-oxo-olean-12-en-28-oic acid (**16**), was characterized by modifications at the C-3 and C-15 carbons, and 7β,15α-dihydroxy-3-oxo-olean-12-en-28-oic acid (**17**), was characterized by modifications at the C-3, C-7 and C-15 carbons.

Ludwig et al. [55] identified two molecules through biotransformation of OA using the bacterium *Nocardia iowensis:* the methyl ester of OA (**18**) and the ketone-methyl ester of OA (**19**) (Table 1). *Circinella muscae AS 3.2695* converted OA at six sites (C-3, C-7, C-12, C-15, C-21, and C-28), producing hydroxylated and glycosylated molecules (**20**–**28**). The derivatives were assessed for anti-inflammatory activity and found to significantly reduce NO generation, with IC_50_ values ranging from 8.28 to 40.74 μM [56]. In a subsequent study, Luchnikova et al. [57] identified two derivatives resulting from the biotransformation of OA by the bacterium *Rhodococcus rhodochrous*. The first molecule has two hydroxyl groups at positions C-5 and C-22 (**29**), as well as two carboxyl groups at position C-23. The second molecule is characterized by a carboxyl group at C-23 (**30**).

## 3. Semi-Synthesis of OA and Biological Activities of Its Derivatives

The discovery of bioactive molecules through organic synthesis remains a persistent challenge. The relationship between synthesis and activity is complex, making the search for compounds with these properties difficult. Therefore, chemists and biologists are working to develop simplified methods for preparing bioactive compounds.

### 3.1. Anti-Cancer Activity 

Throughout history, fatal illnesses have affected the world, with cancer being one of the most significant. In 2018, 18 million people worldwide were affected by cancer, which resulted in 9.6 million deaths [58]. Breast cancer was expected to affect 2.3 million women worldwide in 2020, killing 685,000 of them [48]. OA is recognized as a valuable resource in the search for anti-cancer drugs due to its remarkable activity [22]. Since 2000, researchers have published reports on the synthesis of various derivatives of this acid to combat this disease.

In fact, Yan et al. [59] have synthesized two naturally occurring products from OA and tested their antitumor activity against Hela cells (Table 2). The results indicate that compound **1a** has the highest antitumor activity, with an IC_50_ value of 2.74 μM. Furthermore, Gupta et al. [60] synthesized 13 OA derivatives, composed of ester and amide derivatives, and investigated their antitumor cell growth ability against 9 human tumor cell lines: IMR-32, HOP-62, HCT-15, A-549, SW-620, IGR-OV-1, SF-295, PC-3, and MCF-7. Table 3 demonstrates that the ester compounds exhibited outstanding anticancer properties against IGR-OV-1, while the amide compounds demonstrated good efficacy against HOP-62.

Researchers have conducted extensive studies to create bioactive compounds of OA that aim to reduce side effects. Chen et al. [61] reported that derivatives of OA (Table 4) have strong cytotoxic effects against SMMC-7721. Various hydrophilic compounds were identified in the OA, and their ability to inhibit cancer cell proliferation was evaluated in the MCF-7, PC3, BGC-823, and A549 cell lines (Table 5). Most of the compounds exhibited potent cytotoxic effects. Compound **7a** demonstrated the highest activity (IC_50_ = 0.39 μM) against PC3 cells, while compound **8a** exhibited the highest potency (IC_50_ = 0.22 μM) against A549 cells [62].

Ester derivatives of OA were prepared by Mallavadhani et al. [63], who assessed their antiproliferative efficacy against several cancer cell lines (Table 6). When compared with OA, the in vitro cytotoxic test showed that the majority of the derivatives were effective against lung and SiHa cancer cell lines. Uridine–OA hybrid analogs were prepared and tested for their anti-cancer effects on various human tumor cell lines, including Hep-G2, A549, PC-3, MCF-7 and BGC-823 (Table 7). All synthesized derivatives demonstrated excellent inhibition of proliferation when compared with OA [64]. Recently, Chouaib et al. [65] prepared a series of OA analogues (**12a**–**f**) (Table 8) and assessed their anticancer effects against two cancer lines, SW480 and EMT-6. In addition, they described the cytotoxicity of two series of OA: 1-phenyl-1*H*-[1,2,3]triazol-4-ylmethyl esters (**13a**–**f**) and 1-phenyl-1*H*-[1,2,3]triazol-5-ylmethyl esters (**14a**–**f**) [66]. Li et al. [67] synthesized a number of novel OA compounds that were modified at the C-3 OH position by disulfide, selenium ether, or thioether bonds. The antiproliferative effect of these derivatives was assessed on different types of human cancer cells (HCT116, L02, BEL-7402 and HepG-2) (Table 9). The derivatives containing sulfur ether showed the best antiproliferative effect, especially on BEL-7402 cells. Compared with our acid and the positive reference drug, these OA derivatives showed significantly stronger anti-proliferative effects against these types of cancer cells. Li et al. [68] created novel analogues that target mitochondria (Table 9) in an effort to increase OA’s anticancer properties and therapeutic efficacy. The majority of these analogues were shown to be more powerfully cytotoxic to cancer cells than to normal cells when their efficacy on tumor cell lines was assessed. Compound **16b** was very interesting, as it showed an IC_50_ in A549 cells of 0.81 μM. In further investigation, Şenol et al. [69,70] synthesized two series of new molecules from the natural product OA. The first series comprises OA derivatives in the form of fatty acid esters (**17a** to **17f**), while the second series (**18a** to **18e**) was synthesized from hydrazides and various aromatic aldehydes (Table 10). The cytotoxic properties of the molecules were tested in vitro using the PC3, A549 and BEAS-2B cell lines. In a subsequent study, Şenol et al. [71] synthesized a novel series of OA-derived α-unsaturated ketone derivatives (**19a** to **19i**) with changes in C-2, C-3 and C-28. The compounds were evaluated against PC3 (Table 10). Their results indicate that these analogues are remarkably less toxic to HUVEC when compared with the reference drug doxorubicin.

In another work, Sheng et al. [72] reported four targeted hydrogen sulfide donor–OA hybrids at position C-3 and tested their biological activity, particularly anticancer activity (Table 11). According to the results, a limited number of hybrids showed intermediate inhibition against K562 cell growth. Over time, medicinal chemists have concentrated on developing compounds derived from OA. In a recently published study, Tang et al. [73] synthesized novel OA–dithiocarbamate conjugates and evaluated their biological activity (Table 12). Analogue **22e** demonstrated the strongest and most comprehensive antiproliferative effects, as demonstrated by the test findings. It exhibited strong activity against A549, Hela, Huh-7, Panc1, HT-29, and Hep3B cells. Yu et al. [74] obtained a series of pyrazole-fused analogues of OA (Table 12). These derivatives were based on the pyrazole-fused derivatives of betulinic acid, which have demonstrated strong therapeutic activity. The effects of these molecules were assessed on the RAW264.7 cancer cell line. The strong cytotoxicity observed for some of these provides valuable clues for the development of new anti-tumor agents.

### 3.2. Anti-Diabetic Activity

It has been scientifically established that the liver centrally regulates the body’s glucose balance [75]. Controlling diabetes is crucial, due to its increasing prevalence worldwide. Type 2 diabetes affects a significant proportion of the adult population, estimated to be around 9% in 2014 [76]. 

In this context, several studies have shown that OA is effective in treating diabetes and metabolic syndrome. It is beneficial in improving the response to insulin, which helps to preserve β-cell functionality and survival. Additionally, it offers protection against the complications of this chronic disease [77]. Ali et al. [78] conducted one of the earliest studies to demonstrate the anti-diabetic effects of OA. The study evaluated the ability of five OA derivatives, which were modified in rings A, C, and D, to inhibit urease, *α*-glucosidase, *β*-lactamase, and acetylcholinesterase. The evaluated products had a significant effect on *α*-glucosidase, but no effect on other enzymes. Compound **24c** demonstrated the highest potency as an inhibitor of this particular enzyme, with an IC_50_ of 7.97 μM. Chen et al. [79] synthesized various structurally diverse compounds of OA, with modifications at ring A (C-3 OH) or ring C (C-28 COOH) and assessed their effects on GPa inhibition. Derivative **25b** exhibited greater potency against this enzyme, demonstrating an IC_50_ value of 3.30 μM. PTP1B is a significant regulator of the insulin pathway, making it a promising target for diabetes control. Based on this information, Zhang et al. [80] prepared various OA modifications at the C-3- and C-28- positions and evaluated their impact on PTP1B. The study found that many of these molecules have a considerable effect on diabetes. The previous study by Cheng et al. [81] focused mainly on the use of click chemistry. They prepared a series of novel nucleoside conjugates of OA and assessed their anti-diabetic activity using the GPa enzyme inhibition assay. They then prepared derivatives of OA dimers and evaluated them against GPa. 

Cheng et al. [82] conducted research by synthesizing derivatives of OA dimers and evaluated their effects against GPa. Their study determined that analogue **30** was the most effective, showing an IC_50_ of 2.59 μM. In order to investigate the potential inhibitory effects of OA derivatives on PTP1B, a series of derivatives were synthesized with modifications to the carboxyl (C-28) and hydroxyl (C-3) groups. Compound **31f** exhibited the strongest inhibitory activity, with an IC_50_ value of 3.12 µM. A molecular docking study on this molecule revealed that the crucial sites for the inhibitory activity of the PTP1B enzyme are the integrity of the A ring and the 12-ene units. In addition, hydrophilic and acidic groups play an essential role, as does the distance between the oleanene and these acidic groups [83]. Nie et al. [84] developed several OA compounds, focusing on modifications at the C-3 and C-28 sites of the structure. The objective was to design *α*-glucosidase inhibitors that incorporated a piperazine moiety to link the cinnamic acid moiety to OA at C-28. The majority of these new compounds displayed superior *α*-glucosidase inhibition compared with our acid. In particular, compound **33d** showed potent inhibitory properties against this enzyme at an IC_50_ of 1.90 μM. This is about 50 times lower than our lead compound (IC_50_ of 98.50 μM) and 200 times lower than acarbose (IC_50_ of 388.00 μM). Zhang et al. [75] demonstrated the anti-diabetic properties of new derivatives of OA, which are characterized by modifications at the C-3 site. In addition, all of these derivatives underwent rigorous in vitro biological evaluations using GPa. The results show that several derivatives exhibited medium to substantial anti-glycogen phosphorylase inhibitory effects. Compound **34g** proved particularly interesting, with notable activity (IC_50_ = 5.40 μM) that can be attributed to the presence of the triazole bond and the naphthalene ring. The research carried out by Liu et al. [85] explored a promising method for improving the properties of drugs by altering the carbohydrates in aglycones. They created twenty-four modified versions of OA by adding sugar. The molecules were assessed for their ability to exhibit inhibitory properties against the enzyme PTP1B. Among these, compounds **35a**, **35b**, **35c** and **35d** showed remarkable inhibitory activity against this enzyme. In particular, compound **35c** was the most effective, showing an IC_50_ value of 0.56 μM. In another work, Tang et al. [86] prepared a variety of conjugates using OA and chalcone and evaluated their inhibitory effects. The study indicated that OA derivatives, conjugated with chalcone units in combination with furan, exhibited significant activity compared with other molecules. For instance, molecule **36a** exhibited the most potent inhibitory effect on α-glucosidase, showing an IC_50_ of 3.20 μM.

In previous studies, Zhong et al. [87] focused on triterpenoids, in particular OA, demonstrating a keen interest in these compounds. Structural changes were made at the C-2, 3-OH, 28-COOH, C-12 and C-13 positions to synthesize a number of derivative forms of OA. The derivatives were evaluated for their biological properties in vitro and in vivo, in particular their efficacy against α-glucosidase. The study of the inhibition of this enzyme showed that most of the analogues exhibited significant levels of inhibition. The results highlight that the addition of substituents in the para position on the phenyl ring was particularly beneficial in enhancing the aglucosidase inhibitory activity of the analogue. In their search for new treatments for diabetes, Deng et al. [88] selected and prepared various derivatives of OA oxime esters (**38a**–**38k**) to create inhibitors targeting both *α*-glucosidase and *α*-amylase. Their analysis showed that the large number of compounds evaluated had significant activity against both enzymes. Gao et al. [89] prepared and characterized several new OA analogues modified at the C-2 and C-3 sites by fusion with pyrazole to evaluate their potential as selective inhibitors of *α*-amylase and *α*-glucosidase. The study showed that the novel compound **39d** exhibited potent inhibitory activity against *α*-glucosidase, with an IC_50_ of 2.64 μM. Until now, researchers have focused on finding solutions for type 2 diabetes. Using OA as a starting point, V. Petrova et al. [90] prepared a range of compounds and tested their capacity to inhibit *α*-glucosidase. The derivatives were found to be effective inhibitors of this enzyme (Table 13).

### 3.3. Anti-Inflammatory Activity

**Figure 2:**
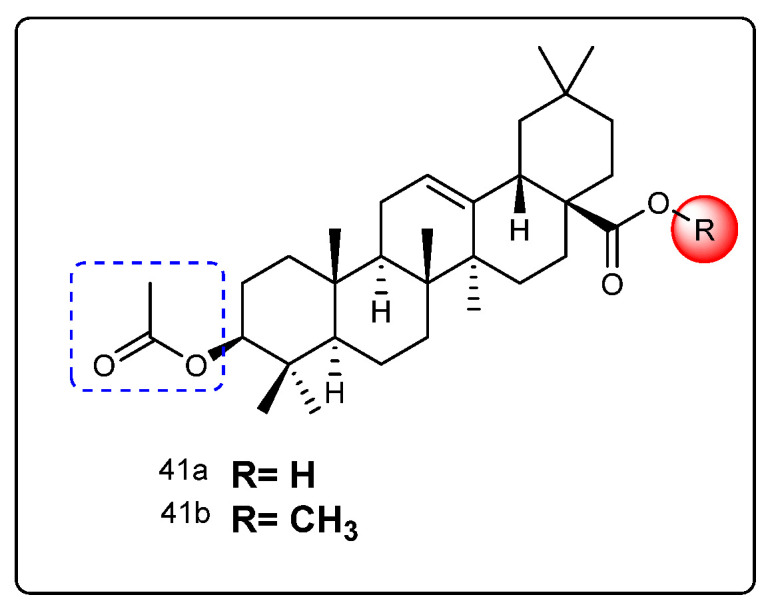
OA analogues.

Anti-inflammatory activity is of paramount importance in medical research, with scientists focusing their efforts on how to reduce the body’s inflammatory responses. Nkeh-Chungag et al. [91] synthesized two derivatives of OA by acetylation and methylation (Figure 2) and evaluated them for anti-inflammatory activity using testing models that cause inflammation through fresh egg albums and serotonin in male Wistar rats. The laboratory also evaluated these compounds for their ability to stabilize erythrocyte membranes in a hemolysis test model induced by heat and low blood pressure. The tests that were carried out showed that the derivatives that were synthesized had more promising anti-inflammatory activity in comparison with the starting molecule.

**Scheme 1:**
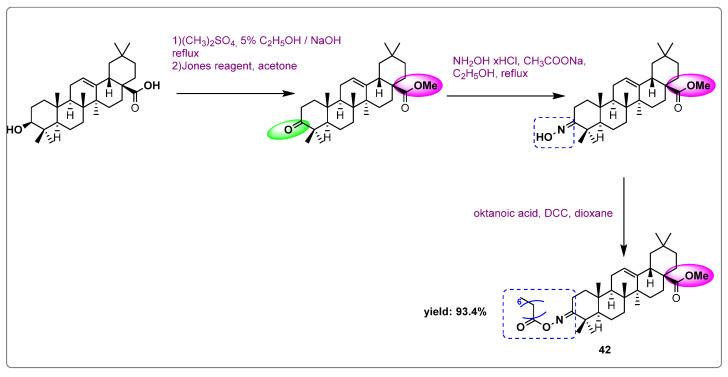
Synthesis of derivatives of methyl 3-octanoyloxyiminoolean-12-en-28-oate [92].

**Scheme 2:**
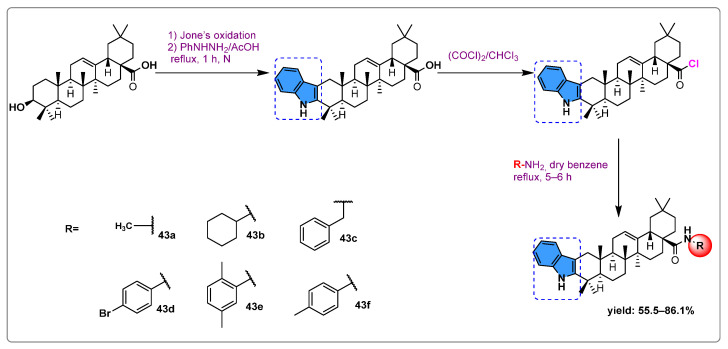
Synthesis of indole derivatives of OA [93].

**Scheme 3:**
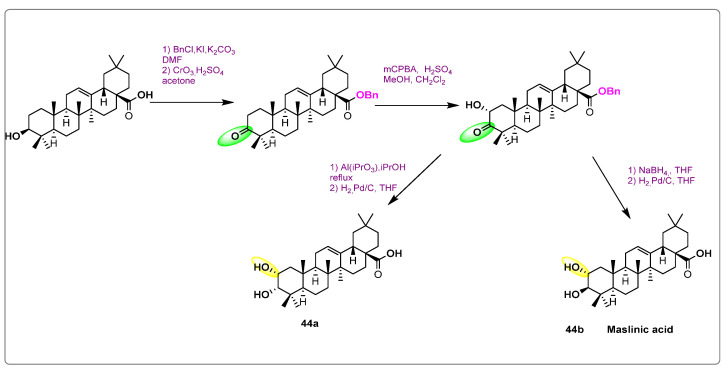
Synthesis of Maslinic acid and 3-epi-maslinic acid [94].

Bednarczyk-Cwynar et al. [92] prepared a methyl-3-octanoyloxyiminoolean-12-en-28-oate derivative of OA and tested it for anti-inflammatory activity (Scheme 1). The evaluation of this molecule involved the administration of carrageenan injections, a substance known to induce significant oedema in the paws of rats. This model is frequently used to investigate the anti-inflammatory properties of different molecules. The synthesized compound exhibited maximum activity between 1.5 and 3.0 h after carrageenan injection. A range of acid derivatives was prepared, characterized by modifications at C-2 and C-3 and leading to the formation of indole-fused derivatives (Scheme 2). These molecules were tested for their anti-inflammatory effects on LPS-induced nitric oxide formation in macrophages. Compared with the NOS inhibitor, these compounds showed a significant impact on NO production, with IC_50_ values ranging from 2.66 to 25.40 μM. Therefore, the prepared OA analogues show enhanced inhibitory activity. According to the studies carried out, the compounds that showed significant activity are characterized by the introduction of a heterocyclic ring in the A cycle of the oleanane skeleton and the insertion of amide groups at C-28 [93]. In a previous study, Nelson et al. [94] demonstrated that maslinic acid and its synthesized derivative exhibit anti-inflammatory activity. This is due to a chemical structural change at the C-2 position of the OA (Scheme 3). The study evaluated two molecules for their potential to inhibit the expression of inflammation-related genes in a mouse model of chemical-induced skin response. Both compounds reduced the expression of inflammatory genes induced by 12-*O*-tetradecanoylphorbol-13 acetate in the skin of the mice. Maslinic acid, though, was stronger than the other compound synthesized.

**Scheme 4:**
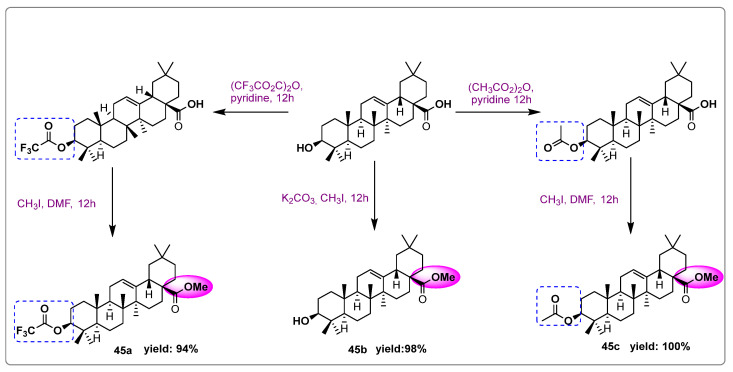
Synthesis of new analogues of OA derivatives [95].

**Scheme 5:**
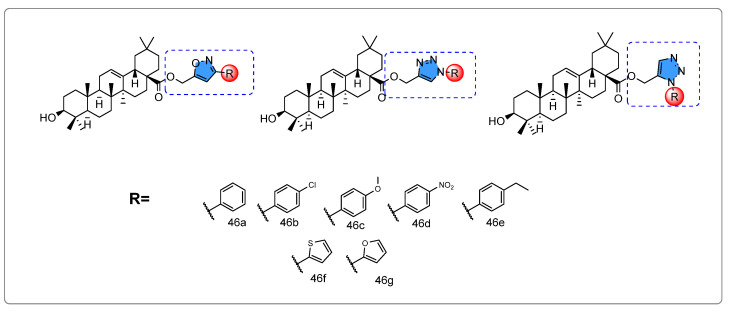
New derivatives of OA–isoxazole and OA–triazole [65,66].

Rali et al. [95] achieved a significant breakthrough by enhancing the anti-inflammatory properties of OA (Scheme 4). They accomplished this by modifying its structure through methylation at the C-28 level of the E ring and acetylation at the C-3 site of the A ring. Isoxazole derivatives of OA were synthesized using the microwave-assisted 1,3-dipolar cycloaddition reaction. The anti-inflammatory properties of the majority of these compounds were studied using PBMCs. This approach allowed for the exploration of the potential of a series of isoxazole derivatives of OA as anti-inflammatory agents [65]. These results encouraged Chouaib [66] to continue his work on OA. He succeeded in synthesizing two series of our acid (Scheme 5). The result of a test using LPS-stimulated PBMCs shows that molecule **46c** has anti-inflammatory activity.

**Scheme 6:**
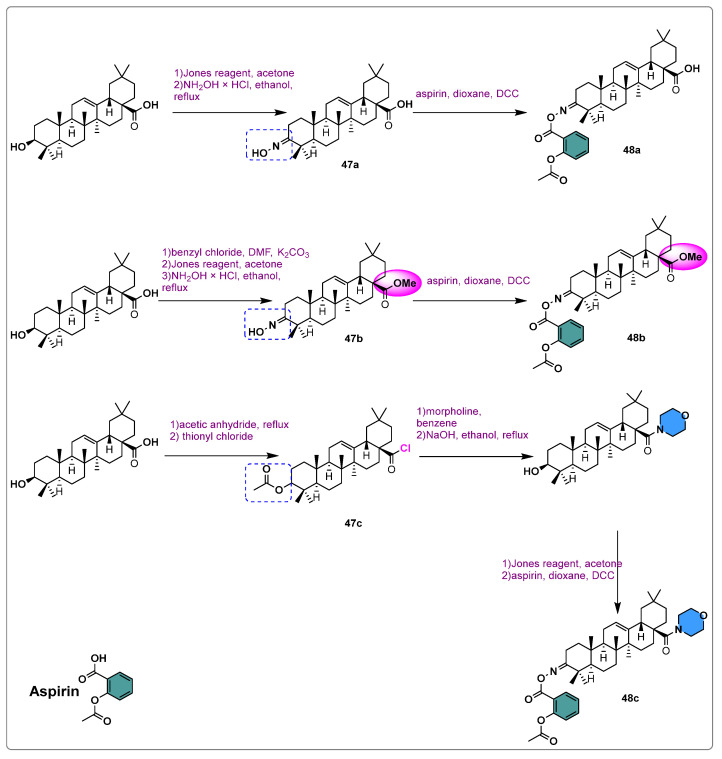
Synthesis of OA–oxime derivatives [96].

**Scheme 7:**
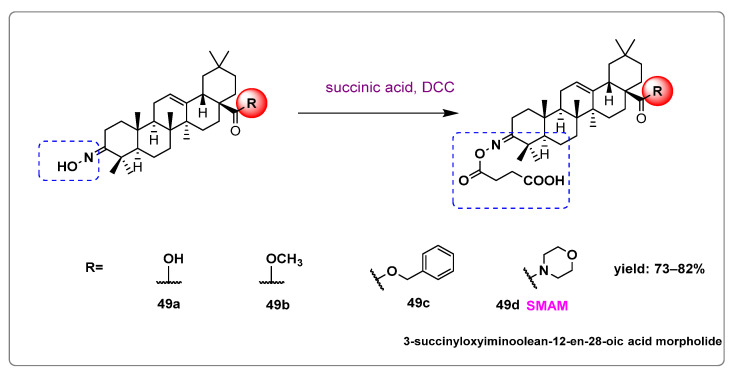
Synthesis of new derivatives of OA–oxime conjugates [97].

In the context of inflammation studies, Krajka-Kuźniak et al. [96] developed new derivatives of OA oxime (Scheme 6) and evaluated their interaction with ASP in modulating NF-κB expression and activation in HepG2 cells, which serve as a human hepatoma model. The results suggest that these derivatives, especially when used with aspirin (derivatives **48a**–**48c**), can affect COX-2 expression in HepG2 cells by regulating the NF-κB pathway. In continuation, Krajka-Kuźniak et al. [97] conducted further research and made structural modifications to the acid compound by incorporating succinic acid at the C-3 site, yielding four novel derivatives of OA oxime (Scheme 7). The derivatives were then tested for their impact on NF-κB and STATs regulation and activation in HepG2 cells. The findings suggest that SMAM is the most potent regulator of both enzymes among the derivatives.

**Scheme 8:**
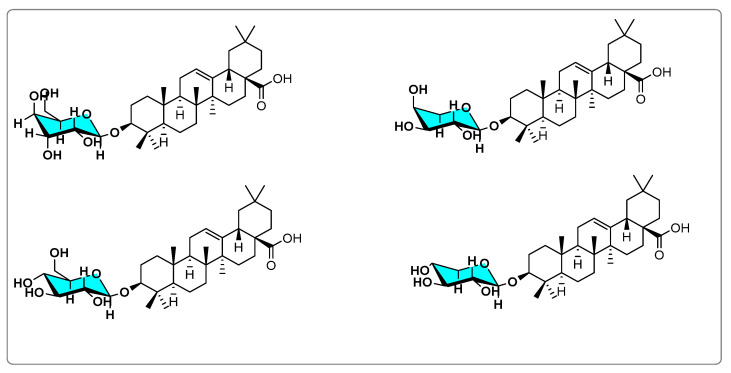
Synthesis of saponin derivatives as agents with anti-inflammatory activity [98].

In another work, Liu et al. [98] synthesized saponin derivatives to enhance the pharmacokinetic properties of OA, aiming to discover more effective anti-inflammatory agents (Scheme 8). In vitro tests have shown that these derivatives greatly inhibit the release of pro-inflammatory factors IL-6 and TNF-α in THP1-derived macrophages activated by LPS.

**Scheme 9:**
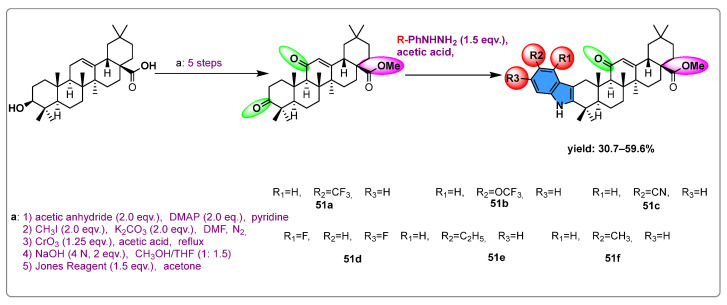
Synthesis of 11 oxo-OA derivatives as agents with anti-inflammatory activity [99].

Jin et al. [99] prepared 11 new analogues of oxooleanolic acid (Scheme 9) to improve its anti-inflammatory activity. Activity was studied using the BV2 cell model of inflammation induced by LPS. In vivo and Western blot studies showed that two derivatives (**51c** and **51d**) significantly inhibited the expression of p-NF-κB, iNOS, p-Akt, p-JNK, p-ERK, p-p38 and COX-2 proteins, while enhancing the expression of HO-1 and Nrf2 proteins in BV2 cells. Both compounds can also exert their anti-inflammatory effects by inhibiting the production of nitric oxide (43.80% and 54.80%), pro-inflammatory cytokines, and chemokines such as MIP-1α, IL-6, TNF-α, IL-12, and IL-1β, while increasing the production of anti-inflammatory cytokines such as IL-10.

**Scheme 10:**
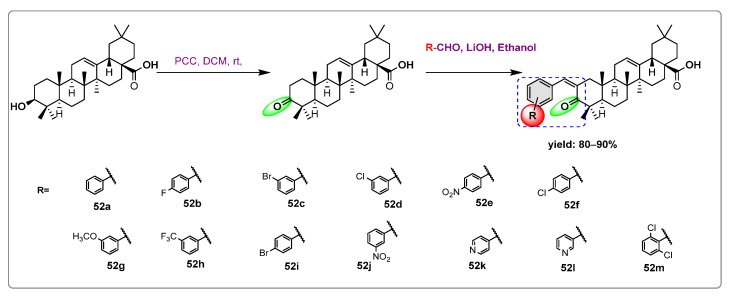
Synthesis of new arylidene derivatives of OA [100].

Hassan Mir et al. [100] synthesized compounds of OA (Scheme 10) and showed anti-inflammatory activity against NO, IL-6 and TNF-α. Altering the C-2 locations of OA’s A ring resulted in the arylidene derivative. These substances have demonstrated stronger anti-inflammatory properties.

### 3.4. Antimicrobial Activity

The emergence of antibiotic resistance in bacteria represents a significant challenge to public health, prompting researchers to explore novel therapeutic strategies. The results of the literature search reveal that several triterpenoids have been demonstrated to possess antimicrobial properties [101]. In particular, oleanolic acid has been identified as a notable example of this phenomenon [102]. The compound has the capacity to inhibit the development of resistance mechanisms in bacteria pathogens [103]. This resistance is achieved through the specific targeting of the bacterial cell envelope [104].

**Scheme 11:**
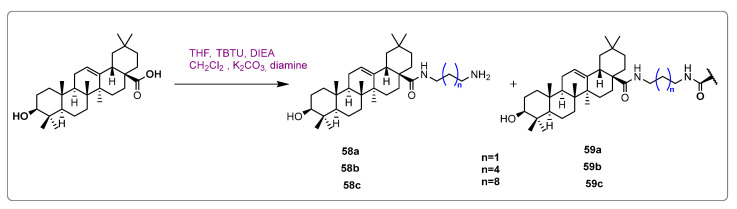
Synthesis of amide analogues with a principal modification at C-28 of OA [103].

Hichri et al. [101] prepared several new derivatives of OA, such as amide, phosphorus, oxidizing and ester compounds (Table 14). The antimicrobial efficacy of these compounds was evaluated on four bacterial strains. The results indicate that compounds **53a** and **53b** showed remarkable efficacy against *Salmonella typhimurium*, which is the most resistant strain. Compounds **53b**, **53c**, **53e**, and **53f** showed moderate efficacy as inhibitors against *Staphylococcus aureus*, *Escherichia coli*, and *Pseudomonas aeruginosa*. Chouaïb et al. [102] prepared a number of OA esters and tested their antimicrobial efficacy against a variety of bacteria, such as *S. aureus* and *E. coli* (Table 15). The study found that OA esters containing sulfur and chlorine atoms show potential as antimicrobial agents. Based on the antimicrobial properties of OA, Blanco-Cabra et al. [103] prepared several amide derivatives modified at C-28 (Scheme 11). These compounds were studied in vivo and in vitro.

**Scheme 12:**
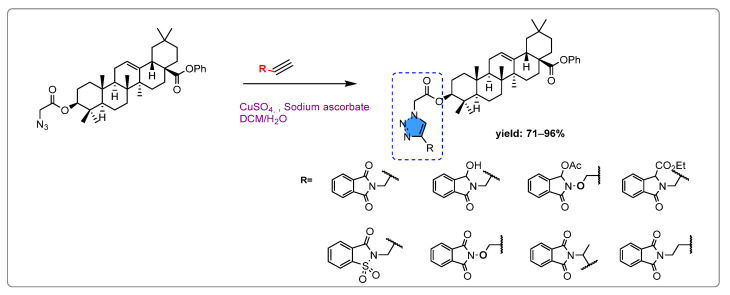
Synthesis of OA–phthalimidine compounds [106].

In a separate study, Khwaza et al. [104] synthesized hybrid compounds based on OA–4-aminoquinoline and tested their antibacterial efficacy on selected bacterial strains. The synthesized compounds demonstrated antibacterial efficacy against the tested bacterial strains (Table 15). A study conducted in vitro examined the antibacterial effects of various synthesized derivatives of OA against four *Staphylococcus species* (Table 14). The study found no significant antibacterial efficacy, however, even at elevated concentrations [105]. Lahmadi et al. [106] prepared a series of novel OA–phthalimidines (Scheme 12) and assessed their antibacterial effectiveness against various bacteria. The derivatives exhibited greater antibacterial efficacy than OA. A molecular docking study highlighted the importance of hydrogen bonds and hydrophobic interactions for this activity.

In another work, Boulila et al. [107] prepared a novel series of analogs of OA and tested their the antibiofilm and antibacterial efficacy in vitro. Their findings indicate that certain derivatives exhibited significant antibacterial activity (Table 15).

**Table 14:** Evaluation of the antibacterial activity of derivatives of OA against several bacteria (MIC and MBC (µM)).

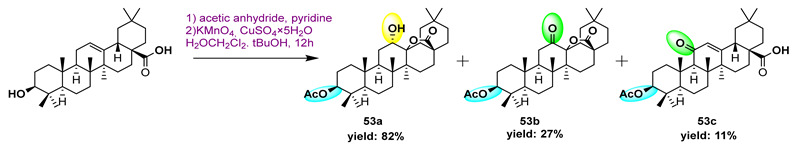							
	**SA** **MIC/MBC**	**EC** **MIC/MBC**	**PA** **MIC/MBC**	**ST** **MIC/MBC**	**SE** **MIC/MBC**	**MS** **MIC/MBC**	**Reference**
**53a** 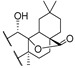	>188/-	>188/-	>/-	126/583	-	-	[101]
**53b** 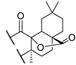	175/1756	175/1756	>189/-	189/585	-	-	
**53c** 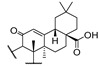	175/975	175/1756	189/1756	175/1756	-	-	
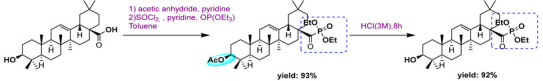							
**53d** 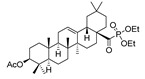	>165/-	>165/-	>165/-	>165 -	-	-	[101]
**53e** 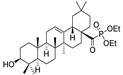	156/1285	156/1653	165/1653	165/1653	-	-	
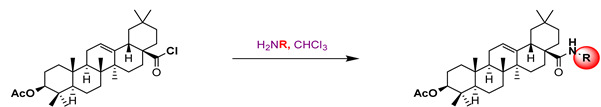							
**53f** 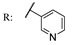	156/1566	156/1566	156/522	156/1218	-	-	[101]
**53g** 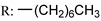	-	-	-	-	-	-	
**53h** 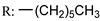	-	-	-	-	-	-	
**53i** 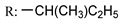	>175/-	>175/-	>175/-	>175/-	-	-	
**53j** 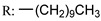	>152/-	>152/-	>152/-	>152/-	-	-	
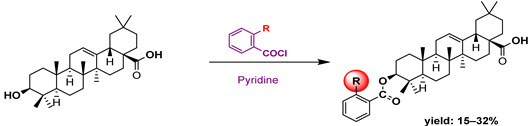							
**53k** 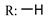	-	-	-	-	-	-	
**53l** 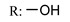	-	-	-	-	-	-	
**53m** 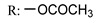	-	-	-	-	-	-	
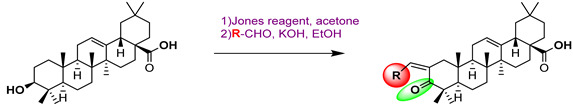							[105]
**54a** 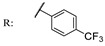	-	-	-	-	>200/-	>200/-	
**54b** 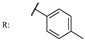	-	-	-	-	>200/-	>200/-	
Gatifloxacin					ND	ND	

SA: Staphylococcus aureus, EC: Escherichia coli, PA: Pseudomonas aeruginosa, ST: Salmonella typhi, SE: Staphylococcus epidermidis, MS: Methicillin-resistant Staphylococcus aureus, MIC: minimum inhibitory concentration, MBC: minimum bacterial concentration, ND: not determined.

**Table 15:** Antibacterial activity of derivatives expressed in MIC and MBC (µM).

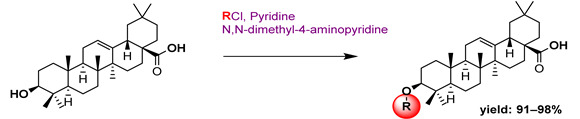									
	**Gram-Positive Bacteria**				** Gram-Negative Bacteria **				** Reference **
	**SA**		**EF**		** EC **		** PA **		[102]
**R**	**MIC**	**MBC**	**MIC**	**MBC**	** MIC **	** MBC **	** MIC **	** MBC **	
**55a** 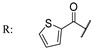	26	52	8	44	8	26	8	26	
**55b** 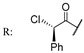	32	164	24	41	8	41	8	49	
**55c** 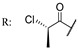	27	45	9	45	9	45	27	45	
**55d** 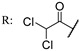	30	52	30	52	8	1.76	8	88	
**55e** 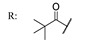	-	-	-	-	-	-	-	-	
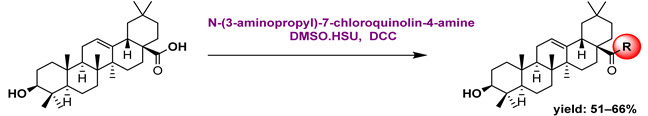									[104]
**56a** 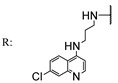	3.71	-	1.85	-	1.85	-	3.71	-	
**56b** 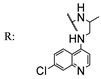	3.71	-	3.71	-	3.71	-	3.71	-	
**56c** 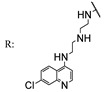	1.77	-	1.77	-	1.77	-	3.55	-	
**56d** 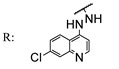	3.95	-	1.97	-	1.97	-	3.95	-	
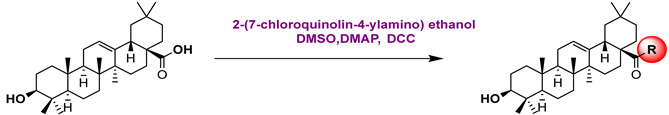									
**56e** 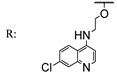	1.89	-	1.89	-	3.78	-	3.78	-	
**56f** 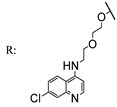	3.54	-	1.77	-	1.77	-	3.54	-	
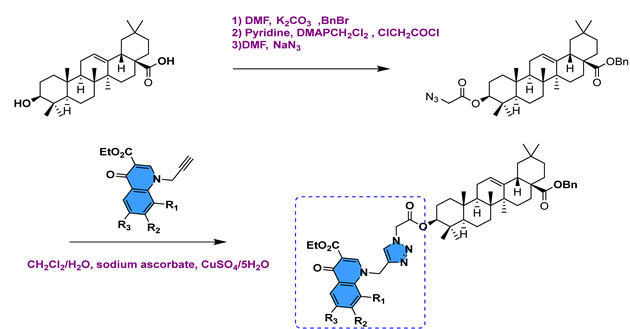									[107]
**57a** 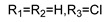	217	-	54	-	217	-	108	-	
**57b** 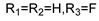	138	-	33.51	-	69.25	-	69.25	-	
**57c** 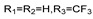	65.61	-	32.80	-	65.61	-	65.61	-	
**57d** 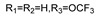	258	-	32.26	-	129	-	32.26	-	
**57e** 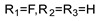	34.62	-	69.25	-	34.62	-	69.25	-	
**57f** 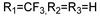	131.23	-	65.61	-	131.23	-	65.61	-	
**57g** 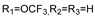	64.53	-	32.26	-	64.53	-	64.53	-	
**57h** 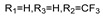	65.61	-	65.61	-	65.61	--	65.61	-	
**57i** 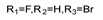	254	-	31.87	-	127	-	31.87	-	
**57j** 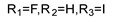	243	-	30.38	-	121	-	30.38	-	
**57k** 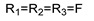	133	-	33.29	-	66.59	--	66.59	-	

SA: *Staphylococcus aureus*, EC: *Escherichia coli*, EF: *Enterococcus faecalis*, MIC: Minimum inhibitory concentration, MBC: minimum bacterial concentration.

### 3.5. Anti-Influenza Activity

**Scheme 13:**
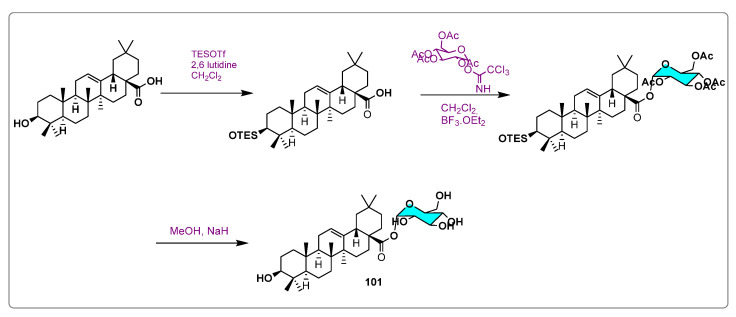
Synthesis of saponin analogue as anti-influenza agent [112].

**Scheme 14:**
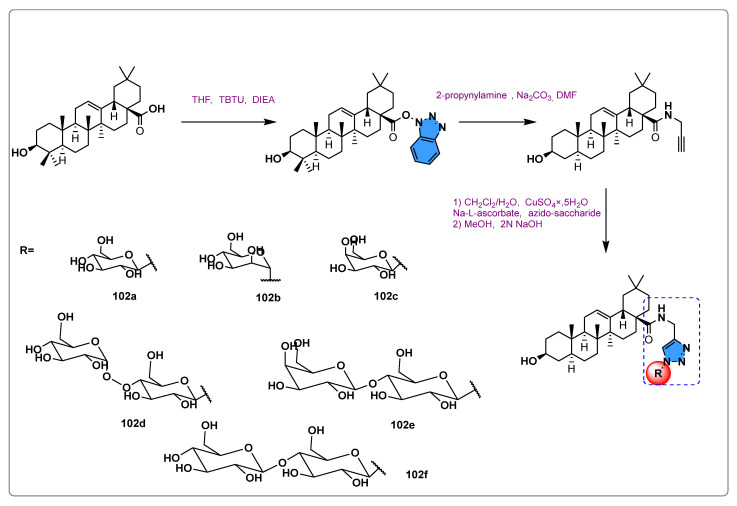
Synthesis of novel sugar-conjugated derivatives of OA [113].

For centuries, medicinal plants have been used to combat disease. However, despite this, human health remains at risk due to the alarming increase in diseases, particularly viral infections, which account for over 65% of all illnesses worldwide [108,109]. Infections with the influenza virus pose a significant threat to human health, resulting in numerous deaths and millions of upper respiratory tract infections each year. It is the most common respiratory pathogen in the world [110,111]. Shirahata et al. [112] discovered a compound of OA to demonstrate its efficacy against viral diseases due to the antiviral activity of the acid (Scheme 13). Their results show that cinnamoyl saponin was an anti-influenza antiviral adjuvant. Su et al. [113] conducted research on the development of OA and evaluated the impact of sugar-conjugated derivatives on anti-influenza activity (Scheme 14). The in vitro studies showed a significant increase in anti-grippal activity of the conjugated compound OA–glucose, with an IC_50_ of 5.47 μM. Broad-spectrum efficacy experiments demonstrated that this compound was effective against both influenza A and B viruses, showing IC_50_ values in the micromole range. This activity is due to the presence of hydrogen bonds and the triazole group.

**Scheme 15:**
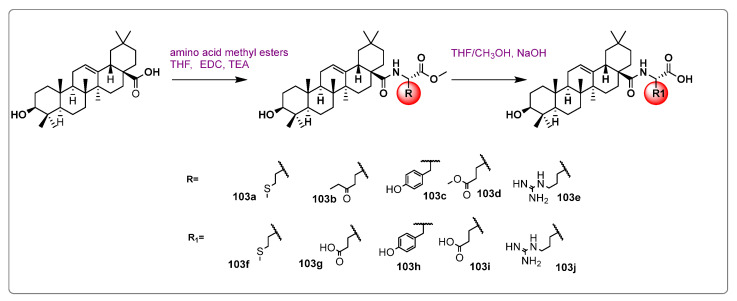
Synthesis of amino derivatives of OA [114].

Meng et al. [114] synthesized derivatives of OA by linking various amino acids to 28-COOH. The aim was to develop molecules that are active against influenza viruses (Scheme 15). The efficacy of these molecules against the Influenza A/WSN/33 (H1N1) virus was studied in vitro. Molecule **103e** showed potent antiviral activity and a broad spectrum of activity with low micromolar IC_50_ values against several influenza variants, including BX-51B, A/WSN/33, BX-35 and A/Texas/50/2012.

**Scheme 16:**
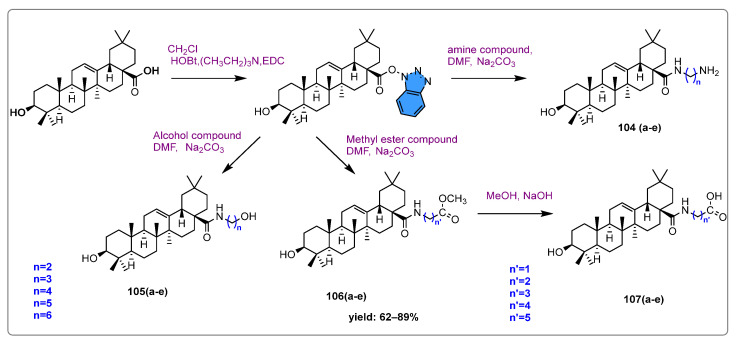
Synthesis of amino derivatives as anti- influenza agents [115].

Previous studies have shown that OA mildly inhibits influenza hemagglutinin (HA). Li et al. [115] prepared a number of several series of OA derivates with structural modifications at C-28 and tested their antiviral efficacy against A/WSN/33 (H1N1) in canine Madin–Darby kidney cells (Scheme 16). Based on the results of the biological assays, compound **105e** exhibited the highest anti-influenza efficacy, with an IC_50_ value of 2.98 µM. This has a six-carbon chain with a terminal hydroxyl group. Furthermore, a surface plasmon resonance assay demonstrated that this derivative can impede influenza virus invasion by significantly interacting with HA protein.

**Scheme 17:**
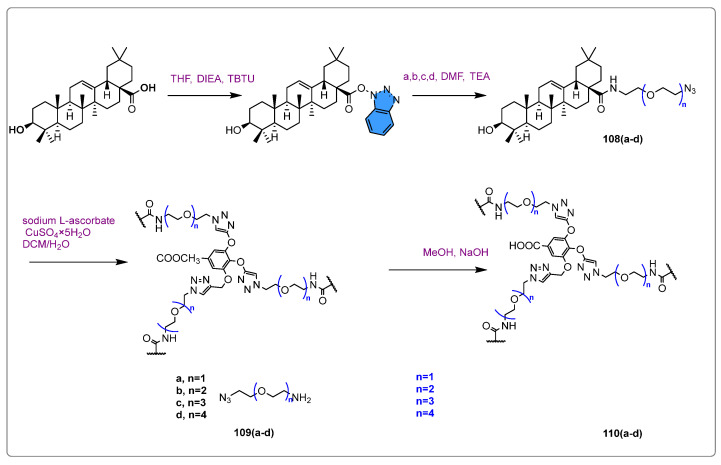
Synthesis of several acid derivatives with potential efficacy against influenza [116].

**Scheme 18:**
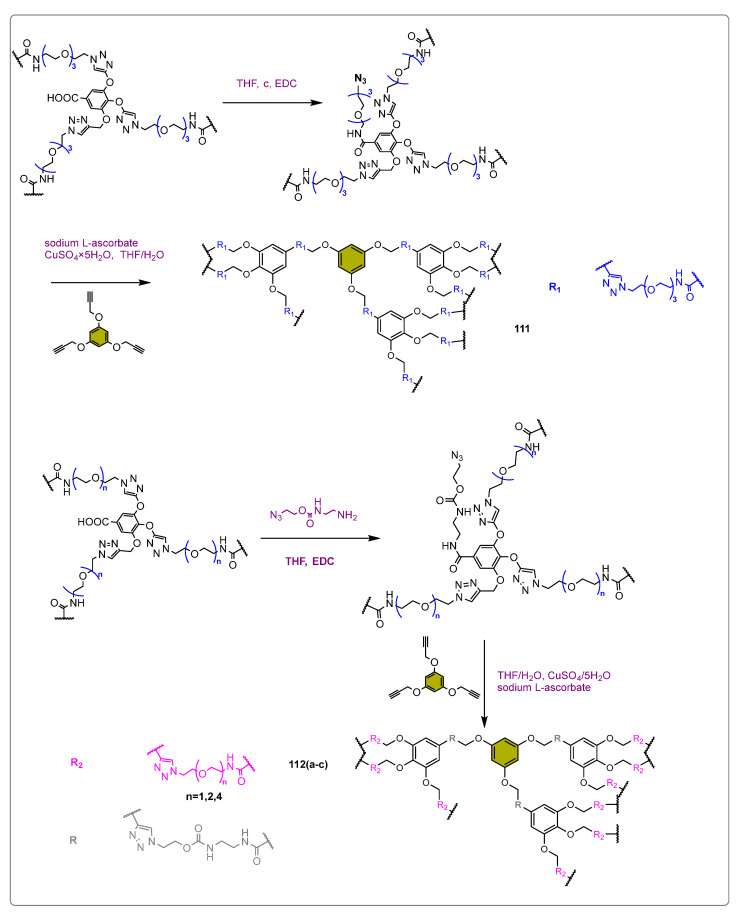
Synthesis of OA derivatives as anti-influenza agents [116].

In a recent study, Shao et al. [116] synthesized nonamer–OA using the CuAAC reaction (Scheme 17 and Scheme 18). The antiviral properties of the prepared compounds were assessed against antiviruses A and B in vitro. Their test results indicate that compounds **111** and **112a** (*n* = 1) had higher IC_50_ values, with compound **111** IC_50_ = 5.23 μM and compound **112a** IC_50_ = 7.93 μM.

### 3.6. Hepatitis Activity

**Scheme 19:**
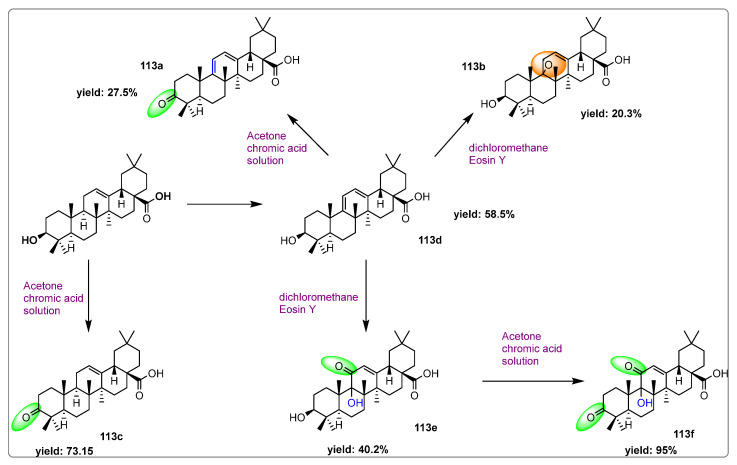
Synthesis of OA derivatives as anti-hepatitis agents [119].

Hepatitis is a disease with a long history that remains a significant global health issue. However, the reason for 10–20% of hepatitis infections is still unknown [117,118]. Li et al. [119] synthesized OA derivatives through various reactions and assessed their efficacy in treating hepatitis (Scheme 19). In vitro and in vivo bioassays demonstrated significant effects, with **113a** exhibiting the most significant activity. Therefore, this molecule has the capability to become a treatment candidate for the hepatitis B virus.

### 3.7. Osteoporosis Activity

**Scheme 20:**
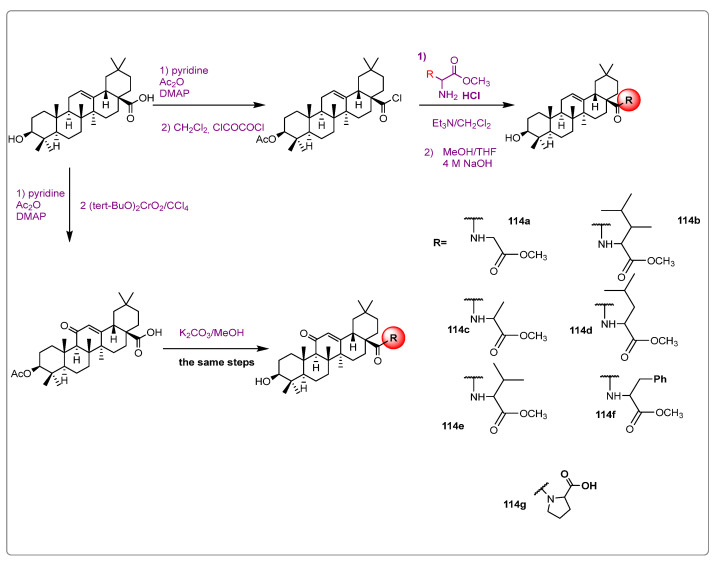
Synthesis of OA derivatives for the treatment of osteoporosis [120].

In the pursuit of new series of molecules, Zhang [120] continued his research on OA to derive other compounds with pharmaceutical properties. Two new series of OA compounds were synthesized by him and by his research team (Scheme 20). Both series were assessed for their capacity to inhibit the formation of MCs produced by vitamin D3 1a,25-dihydroxy. The data suggest that acid derivatives containing phenylalanine and proline have a higher potential for inhibition than both the control (100%) and the amino acids used.

**Scheme 21:**
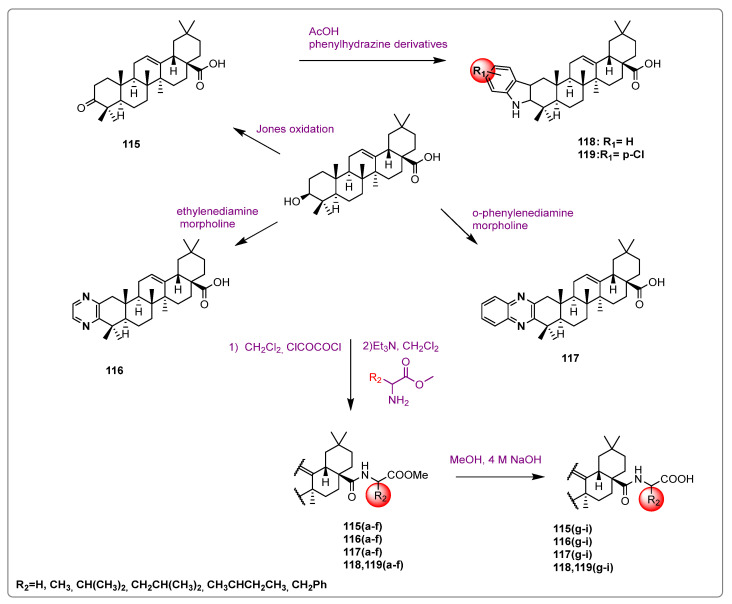
Synthesis of heterocyclic compounds [121].

Li et al. [121] synthesized a number of heterocyclic compounds of OA and tested their inhibition of the production of MCs (Scheme 21). Compounds **115a** and **117** exhibited potent inhibition even at 200 nM. The activity was enhanced by introducing a heterocyclic ring with two nitrogen atoms on the carbonyl group at C-3, according to structure–activity relationships. Additionally, derivatives substituted with glycine and alanine showed improved activity.

**Scheme 22:**
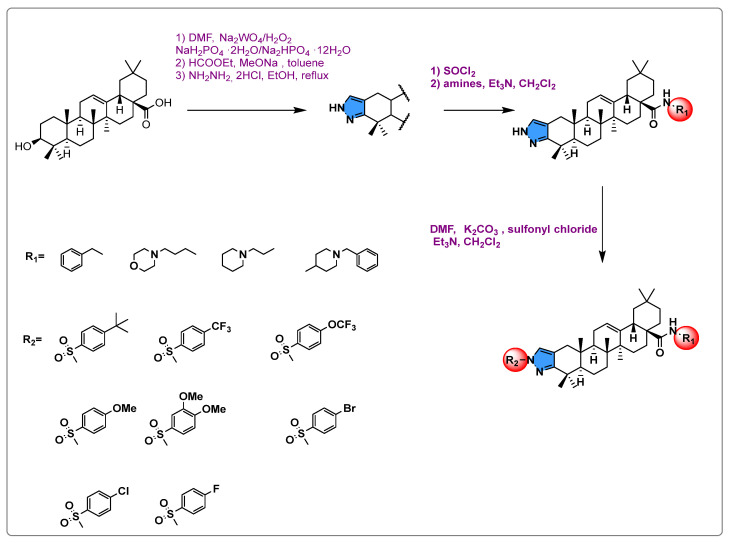
Synthesis of pyrazole compounds [122].

**Scheme 23:**
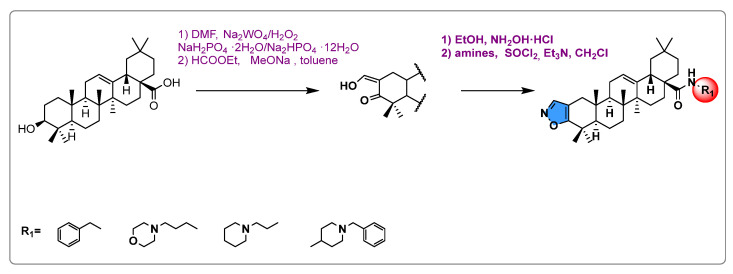
Synthesis of isoxazole compounds [122].

In another work, Wu et al. [122] prepared several heterocyclic analogues, including indole, pyrazine, quinoxaline and quinoline, which were modified on the A ring and C-28 site of our acid (Scheme 22 and Scheme 23). They conducted in vitro tests to determine the anti-bone resorption properties of these derivatives. The screening findings revealed that the majority of the compounds reduced RANKL-induced osteoclast formation from RAW264.7 cells. Furthermore, the pyrazole compounds had better inhibitory activity than the isoxazole compounds.

**Scheme 24:**
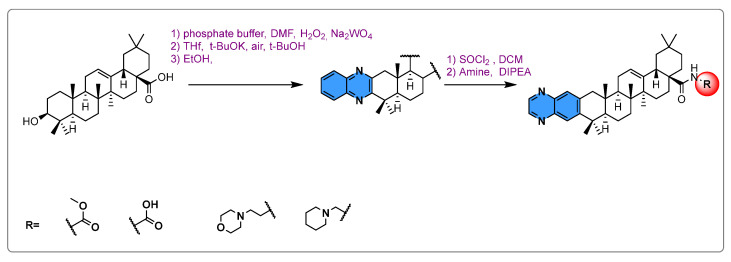
OA compounds as potential osteoclastogenesis inhibitors and anti-osteoporotic agents [123].

In response to the growing prevalence of osteoporosis among the elderly, Zhang et al. [123] investigated a range of compounds with biological activity against the disease (Scheme 24). They synthesized and tested a range of quinoxaline–OA compounds for their inhibitory effect on the nuclear factor kB-induced receptor activator of osteoclastogenesis (RANKL). Their findings indicate that these chemicals could be used as potential leads in the search for new anti-osteoporosis drugs.

## 4. Conclusions

The incorporation of natural compounds into pharmaceutical research is an essential and unavoidable part of the drug development process. OA, with its wide range of pharmacological activities, is currently the focus of extensive research. It offers promising prospects for the treatment of various conditions, including diabetes, cancer, hepatitis, Alzheimer’s disease and viral infections. However, due to the concerning rise in diseases each year and the importance of this acid, it is imperative to search for derivatives. After identifying it as a pharmacological compound, researchers in chemistry and biology undertook structural modifications to improve its efficacy, opening up promising new therapeutic prospects. Previous research has focused mostly on the pharmacological properties and structure–activity correlations of OA and its derivatives. In our study, we examined the structural modifications of OA using organic chemistry and enzymatic approaches. We also evaluated the biological activities of these derivatives and their correlation with their structure, while addressing aspects of organic synthesis.

Our work therefore consists of producing a summary that integrates the chemical and biological aspects of these compounds. It was found that the structural modification of OA primarily focuses on the A, C, and E rings, in conjunction with other bioactive components. Further exploration of biologically active molecules has led to promising results for the study of OA and its derivatives, offering potential relief from psychosomatic diseases. However, our research strategy focuses on broadening the chemical space of OA derivatives and optimizing their therapeutic potential using two complementary approaches: organic chemistry and enzymatic chemistry. Hence, our aim is to improve our understanding of OA and its derivatives, while exploring their potential applications in various biomedical fields.

## Figures and Tables

**Table 1:** Biotransformation of OA by different enzymes and microorganisms.

Names of the Micro-Organisms	Derivatives	Biological Activities	References
*Nocardia* sp. *NRRL 5646*	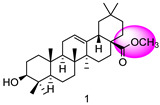	-	[53]
*Furarium lini*	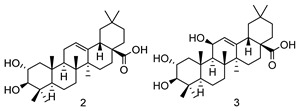	Anti-α-glycosidase activity IC_50_: 2: 444 µM 3: 666 µM	[50]
*Alternaria longipes*	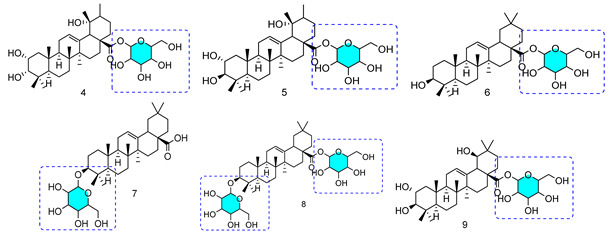	Anticancer activity HeLa Cell Line IC_50_: 4: 7.40 μM	[52]
*Penicillium adametzi*	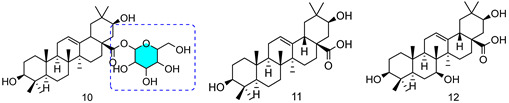	10: 25.00 μM/11: 7.60 μM/12: 0.78 μM	
*Rhizomucor miehei*	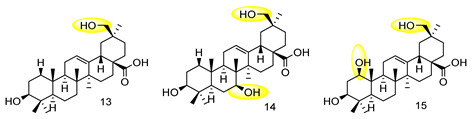	-	[54]
*Trichothecium roseum*	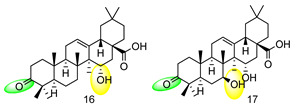	-	[51]
*Nocardia iowensis*	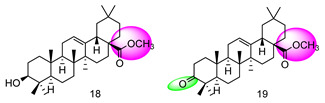	-	[55]
*Circinella muscae*	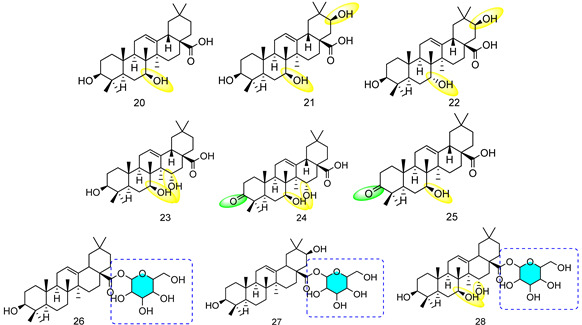	Anti-inflammatory activities Cell: RAW 264.7 IC_50_: 20: 9.24 μM/21: 56.13 μM/22: 68.39 μM 23: 10.06 μM/24: 34.63 μM/25: 39.83 μM 26: 11.28 μM/27: 40.74 μM/28: 8.28 μM	[56]
*Rhodococcus rhodochrous*	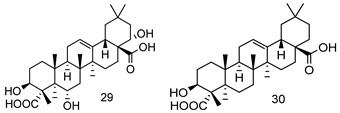	-	[57]

IC_50_: inhibitory concentration required for 50% inhibition.

**Table 2:** Evaluation of natural OA derivatives against the HeLa cell line [59].

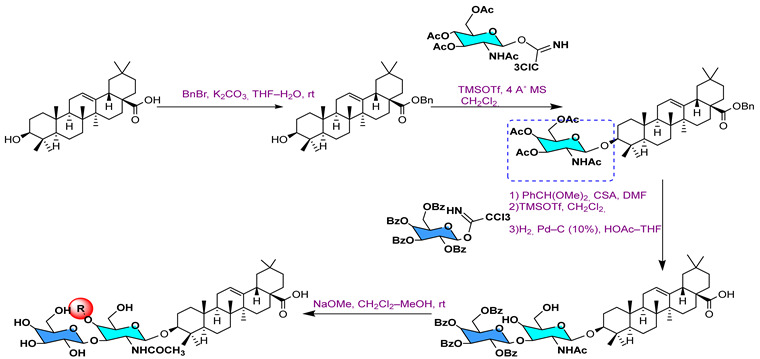	
**1**	**Cell Line Hela IC_50_ (μM)**
**1a** 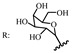	2.74
**1b** 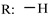	>10

**Table 3:** Evaluation of ester and amide derivatives on two cell lines by inhibition (%) [60].

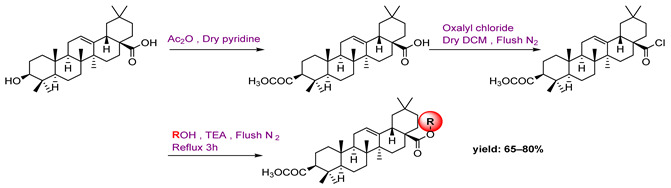		
**2**	**Cell Line: Conc. (50 μM)**	
	**IGR-OV-1**	**HOP-62**
**2a** 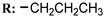	25.00	-
**2b** 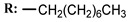	38.00	-
**2c** 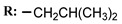	45.00	-
**2d** 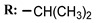	44.00	-
**2e** 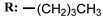	36.00	-
**2f** 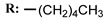	31.00	-
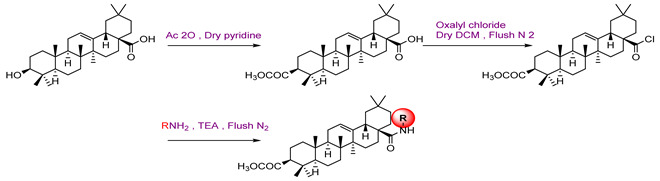 **3**		
**3a** 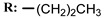	-	22.00
**3b** 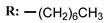	-	13.00
**3c** 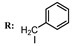	-	27.00
**3d** 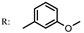	-	53.00
**3e** 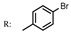	-	32.00
**3f** 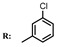	-	22.00
**3g** 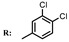	-	16.00

Conc. (M): The molar concentration of the synthesized molecules.

**Table 4:** Evaluation of derivatives against cell viability of various cell lines by inhibition (%) [61].

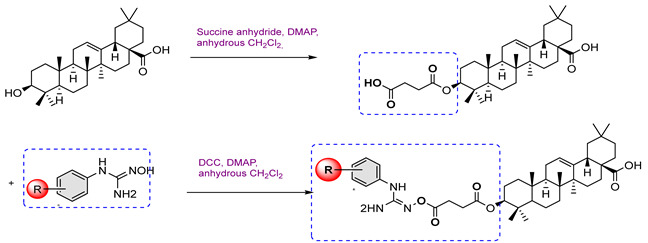				
**4**	**Cell Line: Conc. (10 μM)**			
	**A549**	**HT-29**	**BEL-7402**	**SMMC7721**
**4a** 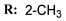	42.30	48.53	47.17	46.63
**4b** 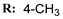	88.43	77.90	73.24	85.86
**4c** 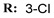	46.15	40.59	40.21	36.53
**4d** 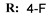	38.99	45.22	35.41	44.80
**4e** 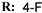	92.30	58.53	77.17	95.26
**4f** 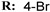	48.90	51.00	45.28	43.75
**4g** 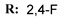	88.43	77.90	73.24	8.86
**4h** 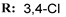	78.64	85.02	65.40	84.80

Conc. (M): The molar concentration of the synthesized molecules.

**Table 5:** Evaluation of derivatives against MCF7 PC-3 A549 BGC-823 [62].

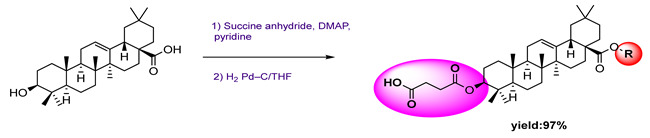				
**5**	**Cell Line IC_50_ (μM)**			
	**MCF7**	**PC-3**	**A549**	**BGC-823**
**5a** 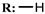	60.70	7.11	6.57	5.59
**5b** 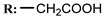	805.00	7.72	11.90	13.40
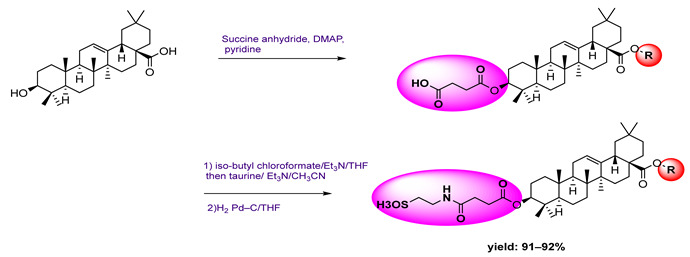 **6**				
**6a** 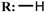	5.19	0.83	1.31	7.32
**6b** 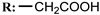	51.70	10.40	268.10	84.50
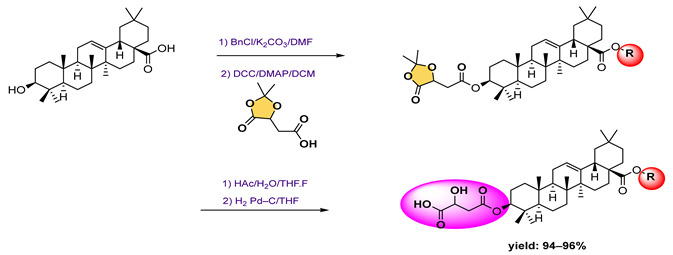 **7**				
**7a** 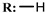	16.20	0.39	0.71	4.18
**7b** 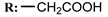	35.10	8.07	19.30	9.36
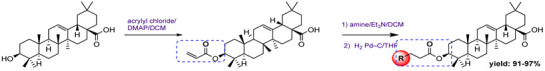 **8**				
**8a** 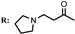	1.98	5.45	6.12	27.20
**8b** 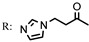	-	50.50	0.22	76.30
**8c** 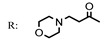	7.18	6.38	727.50	81.90

**Table 6:** Evaluation of ester derivatives against various cell lines by inhibition (%) [63].

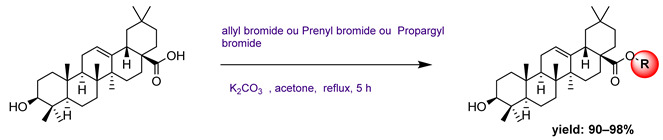						
**9**	**Cell Line: Conc. (50 μM)**					
	**Colo-205**	**SW-620**	**SiHa**	**HeLa**	**A-549**	**IMR-32**
**9a** 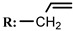	0	1.00	15	0	31.00	16.00
**9b** 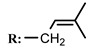	0	13.00	36.00	5.00	25.00	16.00
**9c** 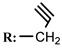	15.00	2.00	30.00	27.00	20.00	16.00

Conc. (M): The molar concentration of the synthesized molecules.

**Table 7:** Evaluation of Uridine–OA hybrid analogues [64].

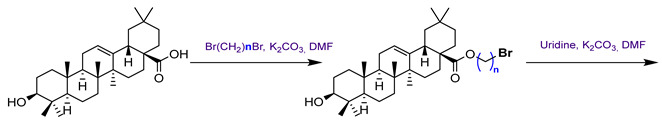 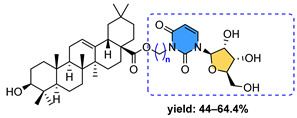					
**10**	**Cell line IC_50_ (μM)**				
	**MCF-7**	**A-549**	**Hep-G2**	**BGC-823**	**PC-3**
**10a** *n* = 2	1.76	2.68	3.92	5.09	1.02
**10b** *n* = 6	2.77	6.04	3.89	3.49	167.46
**10c** *n* = 8	4.45	1.67	3.54	2.24	15.09
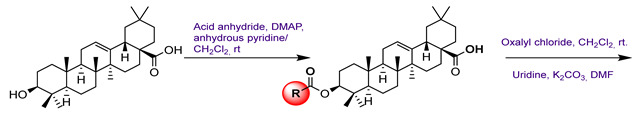 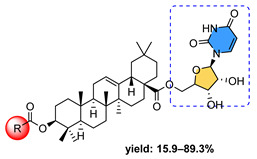 **11**					
**11a** 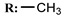	0.07	2.27	4.24	<0.1	2.37
**11b** 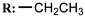	1.06	1.38	4.81	0.14	1.34
**11c** 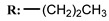	0.83	1.83	5.43	0.29	51.52
**11d** 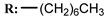	0.85	5.89	4.79	<0.1	2.29
**11e** 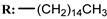	1.85	0.70	4.46	-	1.21
**11f** 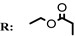	1.48	1.21	3.96	5.91	7.57

**Table 8:** Evaluation of OA derivatives against EMT-6 and SW480.

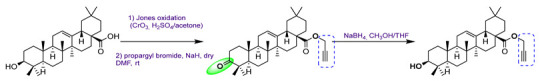 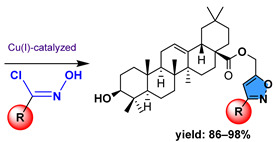			
**12**	**Cell Line Viability (% Control)** **Conc. (100 μM)**		**Reference**
	**EMT-6**	**SW480**	
**12a** 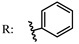	108.00	82.00	[65]
**12b** 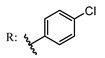	40.00	71.00	
**12c** 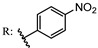	44.00	61.00	
**12d** 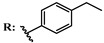	116.00	106.00	
**12e** 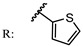	32.00	57.00	
**12f** 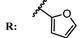	55.00	90.00	
**13** 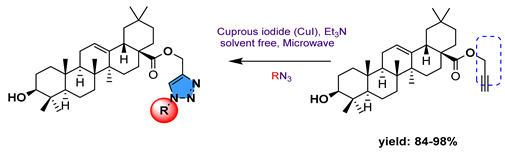 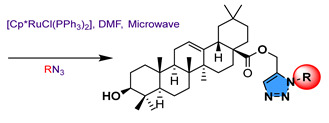 **14**			
**13**–**14a** 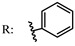	-	-	[66]
**13b** 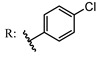 **14b**	42.00 94.00	15.00 102.00	
**13c** 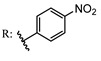 **14c**	-	-	
**13d** 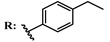 **14d**	78.00 74.00	108.00 105.00	
**13e** 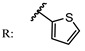	102.00 60.00	112.00 80.00	
**14e**			
**13f** 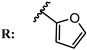	106.00	104.00	

Conc. (M): The molar concentration of the synthesized molecules.

**Table 9:** Antiproliferative activity of derivatives of OA.

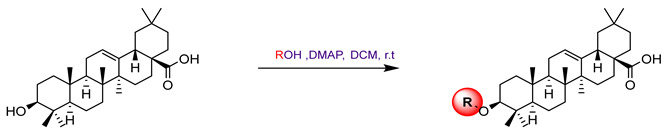					
**15**	**Cell Line IC_50_ (μM)**				**Reference**
	**16 BEL-7**	**HepG2**	**LO2**	**A549**	
**15a** 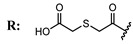	9.81	40.91	26.73	-	[67]
**15b** 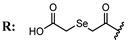	7.08	46.67	30.25	-	
**15c** 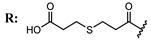	5.70	47.22	25.47	-	
**15d** 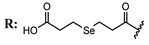	10.12	30.06	13.28	-	
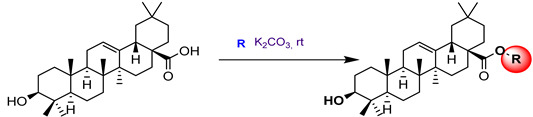 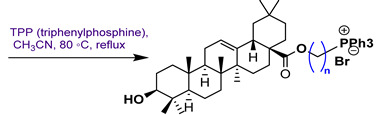 **16**					
**16a** *n* = 3	-	1.06	-	0.93	[68]
**16b** *n* = 5	-	1.32	-	0.81	

**Table 10:** Evaluation of OA derivatives against BEAS-2B, A549 and PC-3.

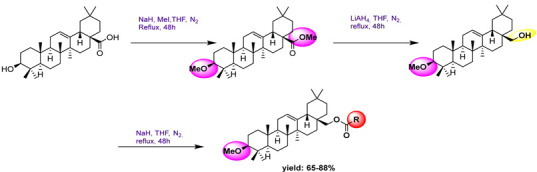				
**17**	**Cell Line: IC_50_ (μM)**			**Reference**
	**BEAS-2B**	**A549**	**PC3**	
**17a** 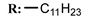	-	-	3.71	[69]
**17b** 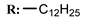	-	-	14.63	
**17c** 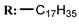	-	-	8.26	
**17d** 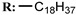	-	-	21.93	
**17e** 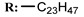	** - **	** - **	15.90	
**17f** 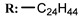	-	-	6.08	
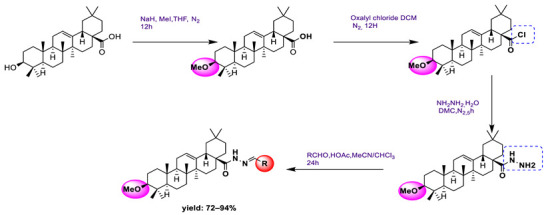 **18**				[70]
**18a** 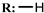	1.15	0.25	-	
**18b** 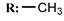	2.96	0.08	-	
**18c** 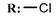	2.76	0.35	-	
**18d** 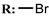	2.31	0.31	-	
**18e** 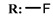	5.26	1.72	-	
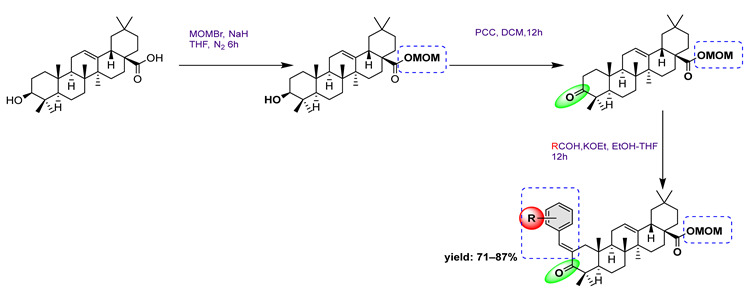 **19**				[71]
**19a** 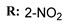	-	-	17.08	
**19b** 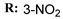	-	-	7.87	
**19c** 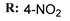	-	-	8.86	
**19d** 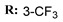	-	-	9.90	
**19e** 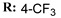	-	-	8.76	
**19f** 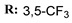	-	-	17.08	
**19g** 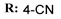	-	-	13.92	
**19h** 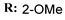	-	-	10.90	
**19i** 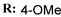	-	-	17.62	

**Table 11:** Anticancer potential of agents of OA against K562 and K562/ADR.

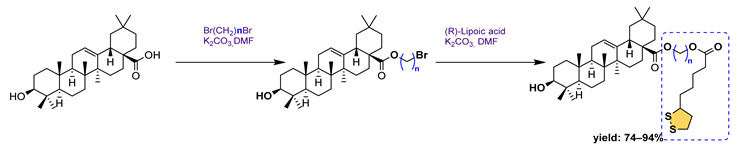 **20**			
	**Cell Line IC_50_ (µM)**		**Reference**
	**K562**	**K562/ADR**	
			[72]
**20a** *n* = 2	89.63	>200	
**20b** *n* = 4	92.39	>200	
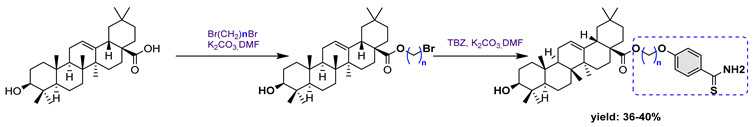 **21**			
**21a** *n* = 6	55.20	74.60	
**21b** *n* = 8	95.00	181.00	

**Table 12:** Evaluation of anti-cancer activity of OA-dithiocarbamate conjugates.

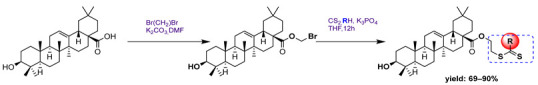 **22**								
	**Cell Line IC_50_ (µM)**							**Reference**
	**A549**	**Hep3B**	**Huh-7**	**HT-29**	**Hela**	**LO2**	**RAW264.7**	
								[73]
**22a** 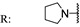	92.10	>200	144.90	-	89.40	113.40	-	
**22b** 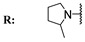	135.80	>200	>200	100.30	77.10	136.00	-	
**22c** 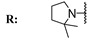	64.30	-	>200	>200	133.70	-	-	
**22d** 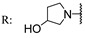	42.50	26.30	64.60	18.30	11.90	34.10	-	
**22e** 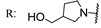	28.80	15.20	29.90	17.60	7.00	62.80	-	
**22f** 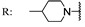	-	176.50	96.90	106.7	106.20	>200	-	
**22g** 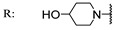	24.40	18.70	70.60	18.4	7.80	30.30	-	
**22h** 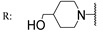	33.60	16.90	49.40	7.60	10.90	25.20	-	
**22i** 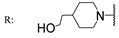	>200	>200	106.70	>200	49.80	>200	-	
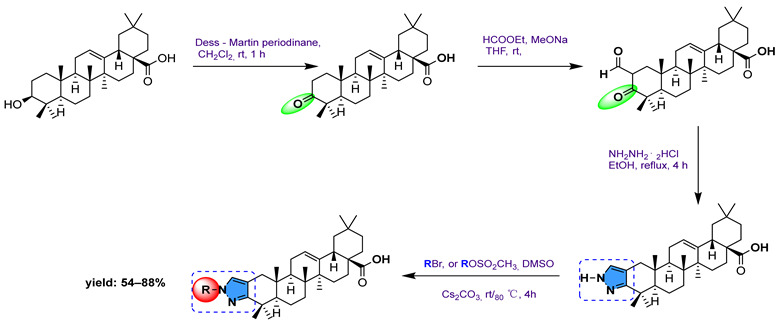 **23**								[74]
**23a** 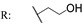	-	-	-	-	-	-	-	
**23b** 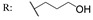	-	-	-	-	-	-	-	
**23c** 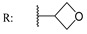	-	-	-	-	-	-	27.30	
**23d** 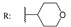	-	-	-	-	-	-	40.45	
**23e** 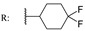	-	-	-	-	-	-	39.28	
**23f** 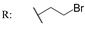	-	-	-	-	-	-	2.67	
**23g** 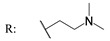	-	-	-	-	-	-	9.17	
**23h** 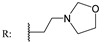	-	-	-	-	-	-	9.31	
**23i** 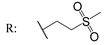	-	-	-	-	-	-	28.30	

**Table 13:** Evaluation of anti-diabetic activity of OA derivatives against various enzymes.

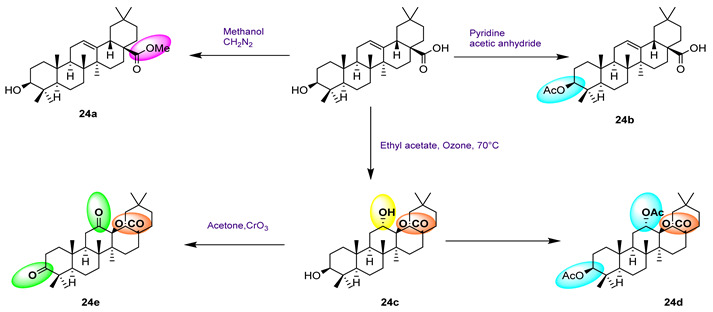						
**24**	**IC_50_ (μM)**					**Reference**
	**α-Glucosidase**	**α-Amylase**	**GPa**	**PTP1B**	**Positive Control**	
**24a** 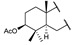	55.09				Deoxynojirimycin: 330.00	[78]
**24b** 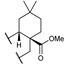	19.01					
**24c** 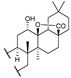	7.97					
**24d** 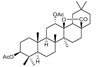	89.71					
**24e** 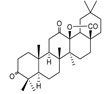	21.63					
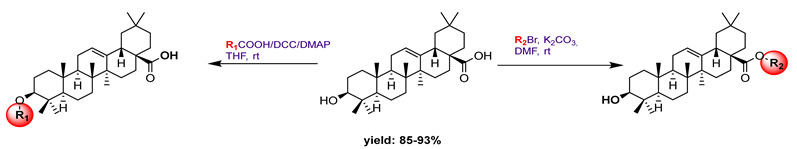 **25**						
**25a** R_1:_ 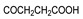 R_2:_ 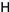			34.30		Caffeine: 114	[79]
**25b** R_1:_ 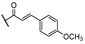 R_2:_ 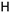			3.30			
**25c** R_1:_ 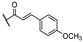 R_2:_ 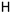			16.90			
**25d** R_1:_ 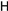 R_2:_ 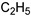			-			
**25e** R_1:_ 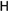 R_2:_ 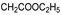			-			
**25f** R_1:_ 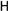 R_2:_ 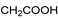			62.60			
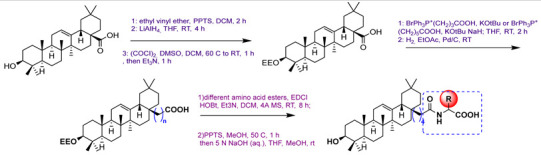 **26**						
**26a** 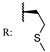				0.55	-	[80]
**26b** 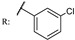				0.51		
**26c** 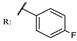				0.57		
**26d** 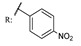				0.45		
**26e** 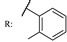				0.55		
**26f** 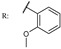				0.53		
**26g** 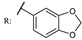				0.44		
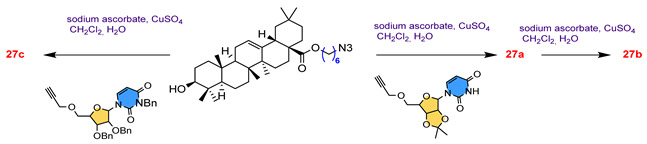 **27**						
**27a** 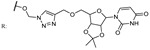			35.00		Caffeine: 98.50	[81]
**27b** 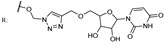			15.50			
**27c** 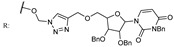			85.50			
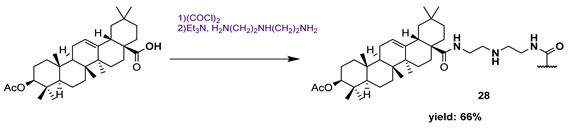					Caffeine: 102.30	[82]
**28**			3.25			
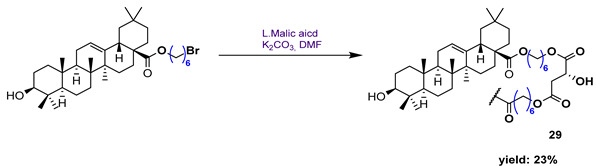						
**29**			12.30			
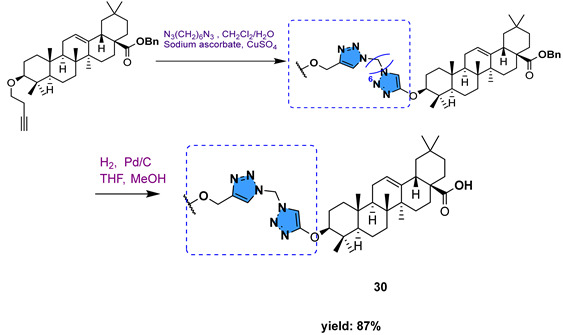						
**30**			2.59			
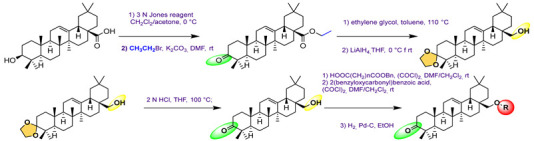 **31**						[83]
**31a** 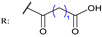				4.11	Sodium Orthovanadate: -	
**31b** 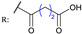				4.61		
**31c** 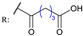				6.39		
**31d** 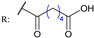				-		
**31e** 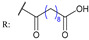				-		
**31f** 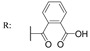				3.12		
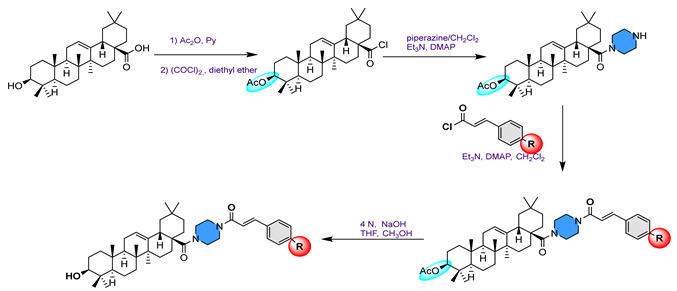 **32**						[84]
**32a** 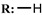	92.60				Acarbose: 388.100	
**32b** 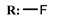	149.70					
**32c** 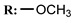	130.00					
**32d** 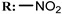	35.50					
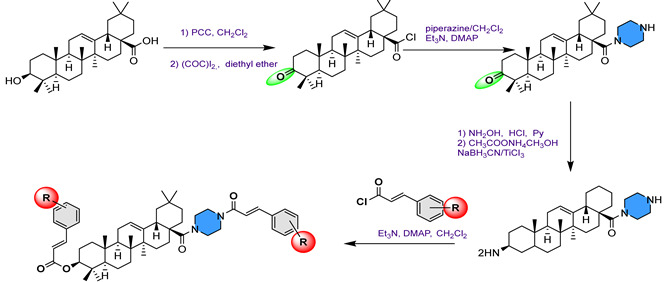 **33**						
**33a** 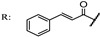	5.90				Acarbose: 388.100	
**33b** 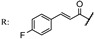	19.70					
**33c** 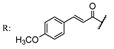	7.90					
**33d** 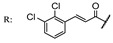	1.90					
**33e** 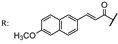	3.90					
**33f** 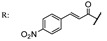	5.90					
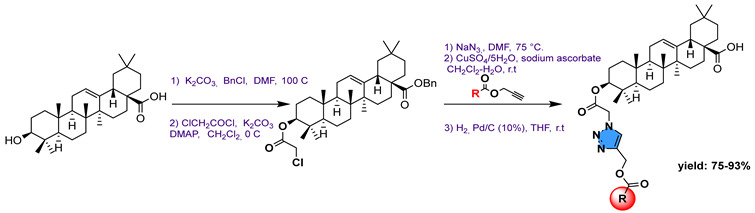 **34**						
**34a** 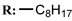			77.10		Caffeine: 144.00	[75]
**34b** 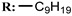			25.60			
**34c** 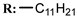			8.40			
**34e** 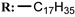			11.20			
**34f** 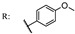			34.60			
**34g** 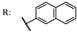			5.40			
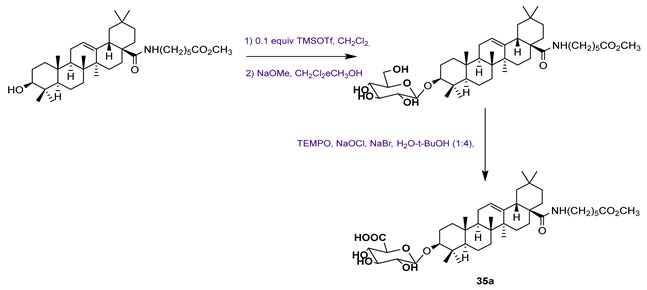						[85]
**35a**				1.91	Sodium Orthovanadate: 8.54	
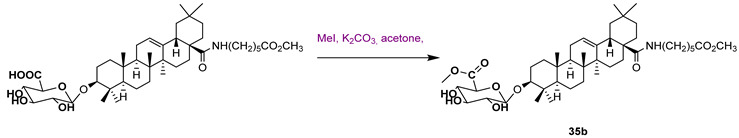						
**35b**				12.20		
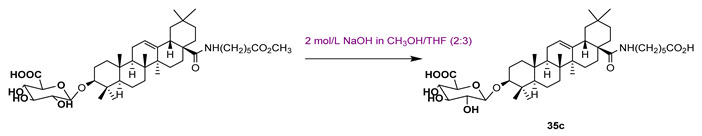						
**35c**				0.56		
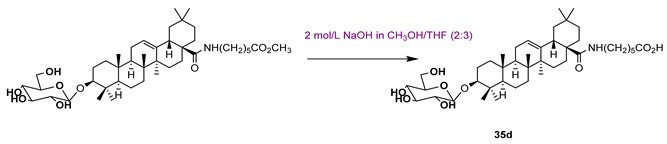						
**35d**				9.21		
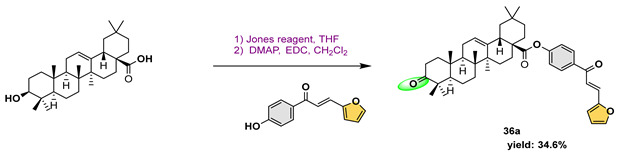						[86]
**36a**	3.20				Acarbose: >300.00	
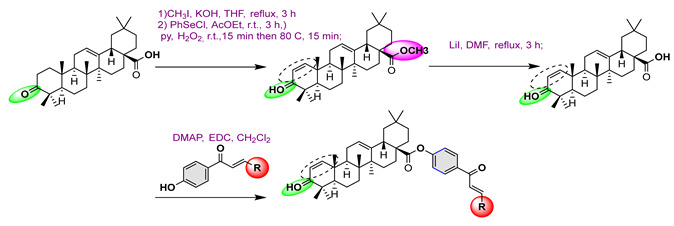						
**36b** 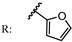	76.90					
**36c** 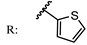	13.50					
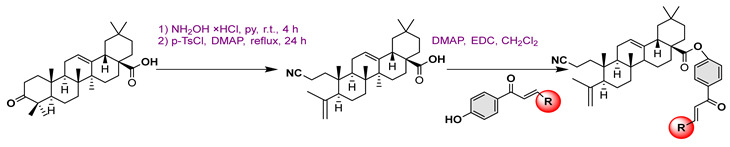						
**36d** 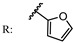	4.10					
**36e** 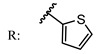	11.50					
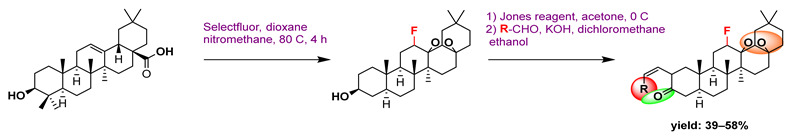 **37**						[87]
**37a** 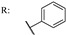	3.43				Acarbose: 579.15	
**37b** 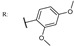	0.98					
**37c** 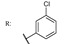	0.72					
**37d** 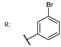	1.29					
**37e** 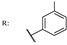	2.74					
**37f** 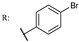	3.29					
**37g** 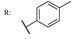	0.33					
**37h** 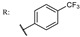	1.74					
**37i** 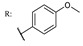	0.69					
**37j** 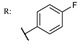	1.92					
**37k** 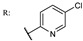	3.67					
**37l** 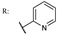	1.17					
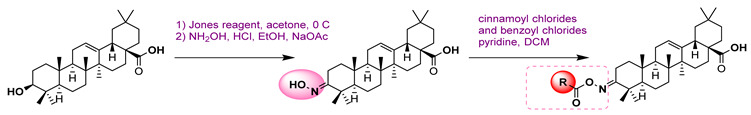 **38**						[88]
**38a** 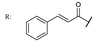	0.35	7.86			Acarbose: 665.56	
**38b** 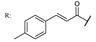	0.68	15.26				
**38c** 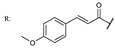	0.89	25.37				
**38d** 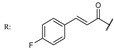	1.45	17.47				
**38e** 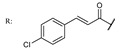	0.95	5.64				
**38f** 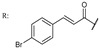	1.28	3.80				
**38g** 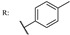	3.13	12.57				
**38h** 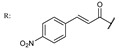	1.36	18.29				
**38i** 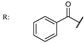	0.67	25.57				
**38j** 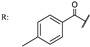	0.99	55.30				
**38k** 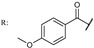	1.10	35.47				
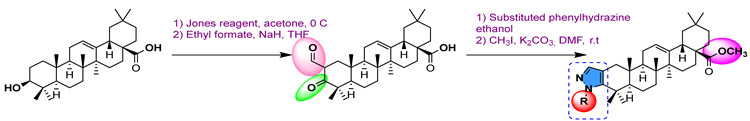 **39**						[89]
**39a** 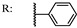	2.44	40.36			Acarbose: 70.82	
**39b** 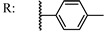	2.60	55.12				
**39c** 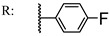	4.51	62.18				
**39d** 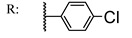	2.64	87.23				
**39e** 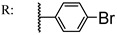	2.79	20.46				
**39f** 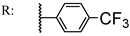	4.86	36.18				
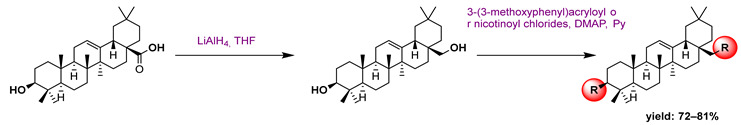 **40**						[90]
**40a** 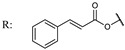	2.40				Acarbose: 436.00	
**40b** 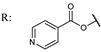	4.56					
**40c** 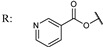	752.60					
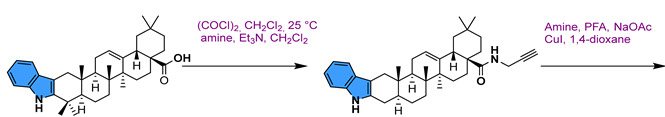 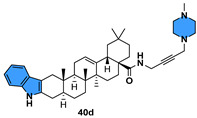						
**40d**	3.01					
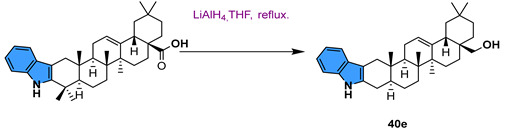						
**40e**	12.37					

## Data Availability

The study did not report any data.

## Abbreviations

OA	Oleanolic acid
TCM	Traditional Chinese Medicine
WHO	World Health Organization
GPa	Rabbit muscle glycogen phosphorylase a
PTP1B	Protein tyrosine phosphatase 1B
LPS	Lipopolysaccharide
NOS	Nitric oxide synthase
NO	Nitric oxide
PBMCs	Human peripheral blood mononuclear cells
ASP	Aspirin
NF-κB	Nuclear factor-κB
COX-2	Cyclooxygenase-2
SMAM	3-succinyloxyiminoolean-12-en-28-oic acid morpholide
TNF-α	Tumor necrosis factor α
IL-6	Interleukin-6
iNOS	Inducible nitric oxide synthase
HO-1	Heme oxygenase-1
Nrf2	Nuclear factor erythroid 2-related factor 2

## References

[1] Che C.T., Zhang H. (2019). Plant Natural Products for Human Health. Int. J. Mol. Sci..

[2] Namdeo P., Gidwani B., Tiwari S., Jain V., Joshi V., Shukla S.S., Pandey R.K., Vyas A. (2023). Therapeutic Potential and Novel Formulations of Ursolic Acid and Its Derivatives: An Updated Review. J. Sci. Food Agric..

[3] Yang Y., Chen K., Wang G., Liu H., Shao L., Zhou X., Liu L., Yang S. (2023). Discovery of Novel Pentacyclic Triterpene Acid Amide Derivatives as Excellent Antimicrobial Agents Dependent on Generation of Reactive Oxygen Species. Int. J. Mol. Sci..

[4] Sinda P.V.K., Tchuenguem R.T., Ponou B.K., Kühlborn J., Kianfé B.Y., Dzoyem J.P., Teponno R.B., Opatz T., Barboni L., Tapondjou L.A. (2022). Antimicrobial Activities of Extract, Fractions and Compounds from the Medicinal Plant *Helichrysum odoratissimun* (L.) Sweet (Asteraceae). S. Afr. J. Bot..

[5] Martial D.E., Dimitry M.Y., Selestin S.D., Nicolas N.Y. (2020). Hypolipidemic and Antioxidant Activity of Aqueous Extract of *Clerodendrum thomsoniae Linn*. (Verbenaceae) Leaves in Albino Rats, *Rattus norvegicus* (Muridae). J. Pharmacogn. Phytochem..

[6] Saleem M., Nazir M., Ali M.S., Hussain H., Lee Y.S., Riaz N., Jabbar A. (2010). Antimicrobial Natural Products: An Update on Future Antibiotic Drug Candidates. Nat. Prod. Rep..

[7] Khwaza V., Oyedeji O.O., Aderibigbe B.A. (2018). Antiviral Activities of Oleanolic Acid and Its Analogues. Molecules.

[8] Ahmad R., Ahmad N., Naqvi A.A., Shehzad A., Al-Ghamdi M.S. (2017). Role of Traditional Islamic and Arabic Plants in Cancer Therapy. J. Tradit. Complement. Med..

[9] Yi Y., Li J., Lai X., Zhang M., Kuang Y., Bao Y.-O., Yu R., Hong W., Muturi E., Xue H. (2022). Natural Triterpenoids from Licorice Potently Inhibit SARS-CoV-2 Infection. J. Adv. Res..

[10] Khusnutdinova E.F., Sinou V., Babkov D.A., Kazakova O., Brunel J.M. (2022). Development of New Antimicrobial Oleanonic Acid Polyamine Conjugates. Antibiotics.

[11] Gudoityte E., Arandarcikaite O., Mazeikiene I., Bendokas V., Liobikas J. (2021). Ursolic and Oleanolic Acids: Plant Metabolites with Neuroprotective Potential. Int. J. Mol. Sci..

[12] Woźniak L., Szakiel A., Glowacka A., Rozpara E., Marszalek K., Skąpska S. (2023). Triterpenoids of Three Apple Cultivars—Biosynthesis, Antioxidative and Anti-Inflammatory Properties, and Fate during Processing. Molecules.

[13] Hill R.A., Connolly J.D. (2013). Triterpenoids. Nat. Prod. Rep..

[14] Baev D.S., Blokhin M.E., Chirkova V.Y., Belenkaya S.V., Luzina O.A., Yarovaya O.I., Salakhutdinov N.F., Shcherbakov D.N. (2022). Triterpenic Acid Amides as Potential Inhibitors of the SARS-CoV-2 Main Protease. Molecules.

[15] Hill R.A., Connolly J.D. (2017). Triterpenoids. Nat. Prod. Rep..

[16] Lin X., Zhou Q., Zhou L., Sun Y., Han X., Cheng X., Wu M., Lv W., Wang J., Zhao W. (2023). Quinoa (*Chenopodium quinoa* Willd) Bran Saponins Alleviate Hyperuricemia and Inhibit Renal Injury by Regulating the PI3K/AKT/NFκB Signaling Pathway and Uric Acid Transport. J. Agric. Food Chem..

[17] Mioc M., Mioc A., Prodea A., Milan A., Balan-Porcarasu M., Racoviceanu R., Ghiulai R., Iovanescu G., Macasoi I., Draghici G. (2022). Novel Triterpenic Acid—Benzotriazole Esters Act as Pro-Apoptotic Antimelanoma Agents. Int. J. Mol. Sci..

[18] Ghante M.H., Jamkhande P.G. (2019). Role of Pentacyclic Triterpenoids in Chemoprevention and Anticancer Treatment: An Overview on Targets and Underling Mechanisms. J. Pharmacopunct..

[19] Yu Z., Sun W., Peng W., Yu R., Li G., Jiang T. (2016). Pharmacokinetics in Vitro and in Vivo of Two Novel Prodrugs of Oleanolic Acid in Rats and Its Hepatoprotective Effects against Liver Injury Induced by CCl4. Mol. Pharm..

[20] Pollier J., Goossens A. (2012). Oleanolic acid. Phytochemistry.

[21] Yu J., Cen X., Chen G., Tang M., Mo L., Li J. (2022). iTRAQ-Based Quantitative Proteomic Analysis in Liver of *Pomacea canaliculata* Induced by Oleanolic Acid Stress. Pest Manag. Sci..

[22] Tang Z.Y., Li Y., Tang Y.T., Ma X.D., Tang Z.Y. (2022). Anticancer Activity of Oleanolic Acid and Its Derivatives: Recent Advances in Evidence, Target Profiling and Mechanisms of Action. Biomed. Pharmacother..

[23] Dale M.P., Moses T., Johnston E.J., Rosser S.J. (2020). A Systematic Comparison of Triterpenoid Biosynthetic Enzymes for the Production of Oleanolic Acid in *Saccharomyces cerevisiae*. PLoS ONE.

[24] Sapkota A., Choi J.W. (2022). Oleanolic Acid Provides Neuroprotection against Ischemic Stroke through the Inhibition of Microglial Activation and NLRP3 Inflammasome Activation. Biomol. Ther..

[25] Sen A. (2020). Prophylactic and Therapeutic Roles of Oleanolic Acid and Its Derivatives in Several Diseases. WJCC.

[26] Mo W., Su C., Huang J., Liu J., Chen Z., Cheng K. (2016). Synthesis of Acyl Oleanolic Acid-Uracil Conjugates and Their Anti-Tumor Activity. Chem. Cent. J..

[27] Sun X., Xue S., Cui Y., Li M., Chen S., Yue J., Gao Z. (2023). Characterization and Identification of Chemical Constituents in *Corni fructus* and Effect of Storage Using UHPLC-LTQ-Orbitrap-MS. Food Res. Int..

[28] Reguigui A., Ott P.G., Darcsi A., Bakonyi J., Romdhane M., Móricz Á.M. (2023). Nine-Dimensional Bioprofiles of Tunisian Sages (*Salvia officinalis*, *S. aegyptiaca* and *S. verbenaca*) by High-Performance Thin-Layer Chromatography–Effect-Directed Analyses. J. Chromatogr. A.

[29] Kabbash E.M., Abdel-Shakour Z.T., El-Ahmady S.H., Wink M., Ayoub I.M. (2023). Comparative Metabolic Profiling of Olive Leaf Extracts from Twelve Different Cultivars Collected in Both Fruiting and Flowering Seasons. Sci. Rep..

[30] Karygianni L., Cecere M., Argyropoulou A., Hellwig E., Skaltsounis A.L., Wittmer A., Tchorz J.P., Al-Ahmad A. (2019). Compounds from *Olea europaea* and *Pistacia lentiscus* Inhibit Oral Microbial Growth. BMC Complement. Altern. Med..

[31] Odun-Ayo F., Chetty K., Reddy L. (2022). Determination of the Ursolic and Oleanolic Acids Content with the Antioxidant Capacity in Apple Peel Extract of Various Cultivars. Braz. J. Biol..

[32] Krajewska M., Dopierała K., Prochaska K. (2022). The Biomimetic System of Oleanolic Acid and Oleic Acid at the Air-Water Interface–Interactions in Terms of Nanotechnology-Based Drug Delivery Systems. Membranes.

[33] Xu H., Dai W., Xia M., Guo W., Zhao Y., Zhang S., Gao W., You X. (2023). Expression of PnSS Promotes Squalene and Oleanolic Acid (OA) Accumulation in *Aralia elata* via Methyl Jasmonate (MeJA) Induction. Genes.

[34] Sultana N., Ata A. (2008). Oleanolic Acid and Related Derivatives as Medicinally Important Compounds. J. Enzym. Inhib. Med. Chem..

[35] Marquina S., Maldonado N., Garduño-Ramírez M.L., Aranda E., Villarreal M.L., Navarro V., Bye R., Delgado G., Alvarez L. (2001). Bioactive oleanolic acid saponins and other constituents from the roots of *Viguiera decurrens*. Phytochemistry.

[36] Muthoni D.K., Samarakoon S.R., Piyathilaka P.C., Rajagopalan U., Tennekoon K.H., Ediriweera M.K. (2022). Identification of 3-*O*-α-l-arabinosyl oleanolic acid, a triterpenoid saponin, as a new breast cancer stem cell growth inhibitor. Nat. Prod. Res..

[37] Zhao H., Zhou M., Duan L., Wang W., Zhang J., Wang D., Liang X. (2013). Efficient Synthesis and Anti-Fungal Activity of Oleanolic Acid Oxime Esters. Molecules.

[38] Vite M.H., Sonawane M.I., Vayal P.M. (2023). Oleanolic Acid as a Potential Drug Molecule: A Review. World J. Adv. Res. Rev..

[39] Rahman S., Islam R., Kamruzzaman M., Alam K., Jamal A.H.M. (2011). *Ocimum sanctum* L.: A review of phytochemical and pharmacological profile. Am. J. Drug Discov. Dev..

[40] Eid A., Jaradat N., Elmarzugi N. (2017). A Review of chemical constituents and traditional usage of Neem plant (*Azadirachta indica*). Palest. Med. Pharm. J..

[41] Akram M., Asif M., Naveed A., Shah P.A., Uzair M., Shaheen G., Shamim T., Shah S.M.A., Ahmad K. (2011). *Tribulus terrestris* Linn.: A review article. J. Med. Plants Res..

[42] Long C., Yang J., Yang H., Li X., Wang G. (2016). Attenuation of Renal Ischemia/Reperfusion Injury by Oleanolic Acid Preconditioning via Its Antioxidant, Anti-inflammatory, and Anti-apoptotic Activities. Mol. Med. Rep..

[43] Wang X., Ye X., Liu R., Chen H., Bai H., Liang X., Zhang X., Wang Z., Li W., Hai C. (2010). Antioxidant Activities of Oleanolic Acid in Vitro: Possible Role of Nrf2 and MAP Kinases. Chem. Biol. Interact..

[44] Chakravarti B., Maurya R., Siddiqui J.A., Bid H.K., Rajendran S.M., Yadav P.P., Konwar R. (2012). In Vitro Anti-Breast Cancer Activity of Ethanolic Extract of Wrightia tomentosa: Role of pro-Apoptotic Effects of Oleanolic Acid and Urosolic Acid. J. Ethnopharmacol..

[45] Fernández-Aparicio Á., Correa-Rodríguez M., Castellano J.M., Schmidt-RioValle J., Perona J.S., González-Jiménez E. (2022). Potential Molecular Targets of Oleanolic Acid in Insulin Resistance and Underlying Oxidative Stress: A Systematic Review. Antioxidants.

[46] Mahmoudieh M., Naghavi M.R., Sobri Z.M., Azzeme A.M., Abd-Aziz N., Rahman N.M.A.N.A., Alitheen N.B., Hussin Y., Bahmanrokh G., Baharum N.A. (2024). Biotechnological approaches in the production of plant secondary metabolites for treating human viral diseases: Prospects and challenges. Biocatal. Agric. Biotechnol..

[47] Banarase N.B., Kaur C.D. (2022). Whole Whey Stabilized Oleanolic Acid Nanosuspension: Formulation and Evaluation Study. J. Drug Deliv. Sci. Technol..

[48] Lisiak N., Dzikowska P., Wisniewska U., Kaczmarek M., Bednarczyk-Cwynar B., Zaprutko L., Rubis B. (2023). Biological Activity of Oleanolic Acid Derivatives HIMOXOL and Br-HIMOLID in Breast Cancer Cells Is Mediated by ER and EGFR. Int. J. Mol. Sci..

[49] Huali Y., Minghui D., Hongwei J.I.A., Zhang K., Yang L.I.U., Cheng M., Wei X. (2024). A Review of Structural Modification and Biological Activities of Oleanolic Acid. Chin. J. Nat. Med..

[50] Iqbal Choudhary M., Batool I., Nahar Khan S., Sultana N., Adnan Ali Shah S., Ur-Rahman A. (2008). Microbial Transformation of Oleanolic Acid by *Fusarium lini* and α-Glucosidase Inhibitory Activity of Its Transformed Products. Nat. Prod. Res..

[51] Gong T., Zheng L., Zhen X., He H.-X., Zhu H.-X., Zhu P. (2014). Microbial Transformation of Oleanolic Acid by *Trichothecium roseum*. J. Asian Nat. Prod. Res..

[52] Liu D.-L., Liu Y., Qiu F., Gao Y., Zhang J.-Z. (2011). Biotransformation of Oleanolic Acid by *Alternaria longipes* and *Penicillium adametzi*. J. Asian Nat. Prod. Res..

[53] Zhang J., Cheng Z.-H., Yu B.-Y., Cordell G.A., Qiu S.X. (2005). Novel Biotransformation of Pentacyclic Triterpenoid Acids by *Nocardia* sp. NRRL 5646. Tetrahedron Lett..

[54] Martinez A., Rivas F., Perojil A., Parra A., Garcia-Granados A., Fernandez-Vivas A. (2013). Biotransformation of Oleanolic and Maslinic Acids by *Rhizomucor miehei*. Phytochemistry.

[55] Ludwig B., Geib D., Haas C., Steingroewer J., Bley T., Muffler K., Ulber R. (2015). Whole-cell Biotransformation of Oleanolic Acid by Free and Immobilized Cells of *Nocardia Iowensis*: Characterization of New Metabolites. Eng. Life Sci..

[56] Yan S., Lin H., Huang H., Yang M., Xu B., Chen G. (2019). Microbial Hydroxylation and Glycosidation of Oleanolic Acid by *Circinella muscae* and Their Anti-Inflammatory Activities. Nat. Prod. Res..

[57] Luchnikova N.A., Grishko V.V., Kostrikina N.A., Sorokin V.V., Mulyukin A.L., Ivshina I.B. (2022). Biotransformation of Oleanolic Acid Using *Rhodococcus rhodochrous* IEGM 757. Catalysts.

[58] Gupta N. (2022). A Review on Recent Developments in the Anticancer Potential of Oleanolic Acid and Its Analogs (2017–2020). Mini Rev. Med. Chem..

[59] Yan M.C., Liu Y., Chen H., Ke Y., Xu Q.C., Cheng M. (2006). Synthesis and Antitumor Activity of Two Natural N-Acetylglucosamine-Bearing Triterpenoid Saponins: Lotoidoside D and E. Bioorg. Med. Chem. Lett..

[60] Gupta S., Kalani K., Saxena M., Srivastava S.K., Agrawal S.K., Suri N., Saxena A.K. (2010). Cytotoxic Evaluation of Semisynthetic Ester and Amide Derivatives of Oleanolic Acid. Nat. Prod. Commun..

[61] Chen L., Zhu Z.-F., Meng F., Xu C.-S., Zhang Y.-H. (2010). Synthesis and Cytotoxicity of Oleanolic Acid/N-Aryl-N’-Hydroxyguanidine Hybrids. Chin. J. Nat. Med..

[62] Hao J., Liu J., Wen X., Sun H. (2013). Synthesis and Cytotoxicity Evaluation of Oleanolic Acid Derivatives. Bioorg. Med. Chem. Lett..

[63] Mallavadhani U.V., Mahapatra A., Pattnaik B., Vanga N., Suri N., Saxena A.K. (2013). Synthesis and Anti-Cancer Activity of Some Novel C-17 Analogs of Ursolic and Oleanolic Acids. Med. Chem. Res..

[64] Cheng K., Su C., Huang J., Liu J., Zheng Y., Chen Z. (2016). Conjugation of Uridine with Oleanolic Acid Derivatives as Potential Antitumor Agents. Chem. Biol. Drug Des..

[65] Chouaïb K., Romdhane A., Delemasure S., Dutartre P., Elie N., Touboul D. (2016). Regiospecific Synthesis, Anti-Inflammatory and Anticancer Evaluation of Novel 3,5-Disubstituted Isoxazoles from the Natural Maslinic and Oleanolic Acids. Ind. Crops Prod..

[66] Chouaïb K., Romdhane A., Delemasure S., Dutartre P., Elie N., Touboul D., Jannet H.B. (2019). Regiospecific Synthesis by Copper-and Ruthenium-Catalyzed Azide–Alkyne 1,3-Dipolar Cycloaddition, Anticancer and Anti-Inflammatory Activities of Oleanolic Acid Triazole Derivatives. Arab. J. Chem..

[67] Li Y., Zhang Y., Luan T., Liu C. (2022). Design and Synthesis of Novel Oleanolic Acid-Linked Disulfide, Thioether, or Selenium Ether Moieties as Potent Cytotoxic Agents. Chem. Biodivers..

[68] Li Y., Zeng Q., Wang R., Wang B., Chen R., Wang N., Lu Y., Shi F., Dehaen W., Huai Q. (2022). Synthesis and Discovery of Mitochondria-Targeting Oleanolic Acid Derivatives for Potential PI3K Inhibition. Fitoterapia.

[69] Şenol H., Çokuludağ K., Aktaş A.S., Atasoy S., Dağ A., Topçu G. (2020). Synthesis of New Fatty Acid Derivatives of Oleanane and Ursane Triterpenoids and Investigation of Their in Vitro Cytotoxic Effects on 3T3 Fibroblast and PC3 Prostate Cancer Cell linesLines. Org. Commun..

[70] Halil Ş., Berre M., Büşra Ş.R., Burak K.H., Ebru H. (2022). Synthesis of Oleanolic Acid Hydrazide-Hydrazone Hybrid Derivatives and Investigation of Their Cytotoxic Effects on A549 Human Lung Cancer Cells. Results Chem..

[71] Şenol H., Ghaffari-Moghaddam M., Bulut Ş., Akbaş F., Köse A., Topçu G. (2023). Synthesis and Anticancer Activity of Novel Derivatives of α, β-Unsaturated Ketones Based on Oleanolic Acid: In Vitro and in Silico Studies against Prostate Cancer Cells. Chem. Biodivers..

[72] Sheng L., Huang J., Liu C., Zhang J., Cheng K. (2019). Synthesis of Oleanolic Acid/Ursolic Acid/Glycyrrhetinic Acid-Hydrogen Sulfide Donor Hybrids and Their Antitumor Activity. Med. Chem. Res..

[73] Tang L., Zhang Y., Xu J., Yang Q., Du F., Wu X., Li M., Shen J., Deng S., Zhao Y. (2023). Synthesis of Oleanolic Acid-Dithiocarbamate Conjugates and Evaluation of Their Broad-Spectrum Antitumor Activities. Molecules.

[74] Yu Y., Yuan W., Yuan J., Wei W., He Q., Zhang X., He S., Yang C. (2023). Synthesis and Biological Evaluation of Pyrazole-Fused Oleanolic Acid Derivatives as Novel Inhibitors of Inflammatory and Osteoclast Differentiation. Bioorg. Med. Chem..

[75] Zhang L., Jia X., Dong J., Chen D., Liu J., Zhang L., Wen X. (2014). Synthesis and Evaluation of Novel Oleanolic Acid Derivatives as Potential Antidiabetic Agents. Chem. Biol. Drug Des..

[76] Silva F.S.G., Oliveira P.J., Duarte M.F. (2016). Oleanolic, Ursolic, and Betulinic Acids as Food Supplements or Pharmaceutical Agents for Type 2 Diabetes: Promise or Illusion?. J. Agric. Food Chem..

[77] Castellano J.M., Ramos-Romero S., Perona J.S. (2022). Oleanolic Acid: Extraction, Characterization and Biological Activity. Nutrients.

[78] Ali M.S., Jahangir M., ul Hussan S.S., Choudhary M.I. (2002). Inhibition of α-Glucosidase by Oleanolic Acid and Its Synthetic Derivatives. Phytochemistry.

[79] Chen J., Liu J., Zhang L., Wu G., Hua W., Wu X., Sun H. (2006). Pentacyclic Triterpenes. Part 3: Synthesis and Biological Evaluation of Oleanolic Acid Derivatives as Novel Inhibitors of Glycogen Phosphorylase. Bioorg. Med. Chem. Lett..

[80] Zhang Y.-N., Zhang W., Hong D., Shi L., Shen Q., Li J.-Y., Li J., Hu L.-H. (2008). Oleanolic Acid and Its Derivatives: New Inhibitor of Protein Tyrosine Phosphatase 1B with Cellular Activities. Bioorg. Med. Chem..

[81] Cheng K., Liu J., Sun H., Xie J. (2010). Synthesis of Nucleoside Conjugates as Potential Inhibitors of Glycogen Phosphorylase. Synthesis.

[82] Cheng K., Liu J., Sun H., Xie J. (2010). Synthesis of Oleanolic Acid Dimers as Inhibitors of Glycogen Phosphorylase. Chem. Biodivers..

[83] Qian S., Li H., Chen Y., Zhang W., Yang S., Wu Y. (2010). Synthesis and Biological Evaluation of Oleanolic Acid Derivatives As Inhibitors of Protein Tyrosine Phosphatase 1B. J. Nat. Prod..

[84] Nie W., Luo J.-G., Wang X.-B., Yin H., Sun H.-B., Yao H.-Q., Kong L.-Y. (2011). Synthesis of New α-Glucosidase Inhibitors Based on Oleanolic Acid Incorporating Cinnamic Amides. Chem. Pharm. Bull..

[85] Liu Q.-C., Guo T.-T., Zhang L., Yu Y., Wang P., Yang J.-F., Li Y.-X. (2013). Synthesis and Biological Evaluation of Oleanolic Acid Derivatives as PTP1B Inhibitors. Eur. J. Med. Chem..

[86] Tang C., Zhu L., Chen Y., Qin R., Mei Z., Xu J., Yang G. (2014). Synthesis and Biological Evaluation of Oleanolic Acid Derivative–Chalcone Conjugates as α-Glucosidase Inhibitors. RSC Adv..

[87] Zhong Y.-Y., Chen H.-S., Wu P.-P., Zhang B.-J., Yang Y., Zhu Q.-Y., Zhang C.-G., Zhao S.-Q. (2019). Synthesis and Biological Evaluation of Novel Oleanolic Acid Analogues as Potential α-Glucosidase Inhibitors. Eur. J. Med. Chem..

[88] Deng X.-Y., Ke J.-J., Zheng Y.-Y., Li D.-L., Zhang K., Zheng X., Wu J.-Y., Xiong Z., Wu P.-P., Xu X.-T. (2022). Synthesis and Bioactivities Evaluation of Oleanolic Acid Oxime Ester Derivatives as *α*-Glucosidase and *α*-Amylase Inhibitors. J. Enzym. Inhib. Med. Chem..

[89] Gao M., Ma H., Liu X., Zhang Y., Tang L., Zheng Z., Zhang X., Jiang C., Lin L., Sun H. (2023). Synthesis and Biological Evaluation of Substituted Pyrazole-Fused Oleanolic Acid Derivatives as Novel Selective α-Glucosidase Inhibitors. Chem. Biodivers..

[90] Petrova A.V., Babkov D.A., Khusnutdinova E.F., Baikova I.P., Kazakova O.B., Sokolova E.V., Spasov A.A. (2023). α-Glucosidase Inhibitors Based on Oleanolic Acid for the Treatment of Immunometabolic Disorders. Appl. Sci..

[91] Nkeh-Chungag B.N., Oyedeji O.O., Oyedeji A.O., Ndebia E.J. (2015). Anti-Inflammatory and Membrane-Stabilizing Properties of Two Semisynthetic Derivatives of Oleanolic Acid. Inflammation.

[92] Bednarczyk-Cwynar B., Zaprutko L., Marciniak J., Lewandowski G., Szulc M., Kaminska E., Wachowiak N., Mikolajczak P.L. (2012). The Analgesic and Anti-Inflammatory Effect of New Oleanolic Acid Acyloxyimino Derivative. Eur. J. Pharm. Sci..

[93] Bhandari P., Patel N.K., Gangwal R.P., Sangamwar A.T., Bhutani K.K. (2014). Oleanolic Acid Analogs as NO, TNF-α and IL-1β Inhibitors: Synthesis, Biological Evaluation and Docking Studies. Bioorg. Med. Chem. Lett..

[94] Nelson A.T., Camelio A.M., Claussen K.R., Cho J., Tremmel L., DiGiovanni J., Siegel D. (2015). Synthesis of Oxygenated Oleanolic and Ursolic Acid Derivatives with Anti-Inflammatory Properties. Bioorg. Med. Chem. Lett..

[95] Rali S., Oyedeji O.O., Aremu O.O., Oyedeji A.O., Nkeh-Chungag B.N. (2016). Semisynthesis of Derivatives of Oleanolic Acid from Syzygium Aromaticum and Their Antinociceptive and Anti-Inflammatory Properties. Mediat. Inflamm..

[96] Krajka-Kuźniak V., Bednarczyk-Cwynar B., Paluszczak J., Szaefer H., Narożna M., Zaprutko L., Baer-Dubowska W. (2019). Oleanolic Acid Oxime Derivatives and Their Conjugates with Aspirin Modulate the NF-κB-Mediated Transcription in HepG2 Hepatoma Cells. Bioorg. Chem..

[97] Krajka-Kuźniak V., Bednarczyk-Cwynar B., Narożna M., Szaefer H., Baer-Dubowska W. (2019). Morpholide Derivative of the Novel Oleanolic Oxime and Succinic Acid Conjugate Diminish the Expression and Activity of NF-κB and STATs in Human Hepatocellular Carcinoma Cells. Chem. Biol. Interact..

[98] Liu L., Li H., Hu K., Xu Q., Wen X., Cheng K., Chen C., Yuan H., Dai L., Sun H. (2021). Synthesis and Anti-Inflammatory Activity of Saponin Derivatives of δ-Oleanolic Acid. Eur. J. Med. Chem..

[99] Jin J., He H., Zhang X., Wu R., Gan L., Li D., Lu Y., Wu P., Wong W.-L., Zhang K. (2021). The in Vitro and in Vivo Study of Oleanolic Acid Indole Derivatives as Novel Anti-Inflammatory Agents: Synthesis, Biological Evaluation, and Mechanistic Analysis. Bioorg. Chem..

[100] Hassan Mir R., Godavari G., Siddiqui N.A., Ahmad B., Mothana R., Ullah R., Almarfadi O., Jachak S., Masoodi M. (2021). Design, Synthesis, Molecular Modelling, and Biological Evaluation of Oleanolic Acid-Arylidene Derivatives as Potential Anti-Inflammatory Agents. Drug Des. Devel. Ther..

[101] Hichri F., Jannet H.B., Cheriaa J., Jegham S., Mighri Z. (2003). Antibacterial Activities of a Few Prepared Derivatives of Oleanolic Acid and of Other Natural Triterpenic Compounds. C. R. Chim..

[102] Chouaïb K., Hichri F., Nguir A., Daami-Remadi M., Elie N., Touboul D., Jannet H.B. (2015). Semi-Synthesis of New Antimicrobial Esters from the Natural Oleanolic and Maslinic Acids. Food Chem..

[103] Blanco-Cabra N., Vega-Granados K., Moya-Andérico L., Vukomanovic M., Parra A., Álvarez De Cienfuegos L., Torrents E. (2019). Novel Oleanolic and Maslinic Acid Derivatives as a Promising Treatment against Bacterial Biofilm in Nosocomial Infections: An in Vitro and in Vivo Study. ACS Infect. Dis..

[104] Khwaza V., Oyedeji O.O., Aderibigbe B.A., Morifi E., Fonkui T.Y., Ndinteh D.T., Steenkamp V. (2021). Synthesis, Antibacterial, and Cytotoxicity Evaluation of Oleanolic Acid-4-Aminoquinoline Based Hybrid Compounds. Recent Adv. Anti-Infect. Drug Discov..

[105] Wu P., Tu B., Liang J., Guo S., Cao N., Chen S., Luo Z., Li J., Zheng W., Tang X. (2021). Synthesis and Biological Evaluation of Pentacyclic Triterpenoid Derivatives as Potential Novel Antibacterial Agents. Bioorg. Chem..

[106] Lahmadi G., Horchani M., Dbeibia A., Mahdhi A., Romdhane A., Lawson A.M., Daïch A., Harrath A.H., Ben Jannet H., Othman M. (2023). Novel Oleanolic Acid-Phtalimidines Tethered 1,2,3 Triazole Hybrids as Promising Antibacterial Agents: Design, Synthesis, In Vitro Experiments and In Silico Docking Studies. Molecules.

[107] Boulila B., Horchani M., Duval R., Othman M., Daïch A., Jannet H.B., Romdhane A., Lawson A.M. (2023). Design, Semi-Synthesis and Molecular Docking of New Antibacterial and Antibiofilm Triazole Conjugates from Hydroxy-Triterpene Acids and Fluoroquinolones. New J. Chem..

[108] Sander W.J., O’Neill H.G., Pohl C.H. (2017). Prostaglandin E2 as a Modulator of Viral Infections. Front. Physiol..

[109] Wimmerová M., Bildziukevich U., Wimmer Z. (2023). Selected Plant Triterpenoids and Their Derivatives as Antiviral Agents. Molecules.

[110] Schulz L., Hornung F., Häder A., Radosa L., Brakhage A.A., Löffler B., Deinhardt-Emmer S. (2023). Influenza Virus-Induced Paracrine Cellular Senescence of the Lung Contributes to Enhanced Viral Load. Aging Dis..

[111] Liu Y., Yang L., Wang H., Xiong Y. (2022). Recent Advances in Antiviral Activities of Triterpenoids. Pharmaceuticals.

[112] Shirahata T., Nagai T., Hirata N., Yokoyama M., Katsumi T., Konishi N., Nishino T., Makino K., Yamada H., Kaji E. (2017). Syntheses and Mucosal Adjuvant Activity of Simplified Oleanolic Acid Saponins Possessing Cinnamoyl Ester. Bioorg. Med. Chem..

[113] Su Y., Meng L., Sun J., Li W., Shao L., Chen K., Zhou D., Yang F., Yu F. (2019). Design, Synthesis of Oleanolic Acid-Saccharide Conjugates Using Click Chemistry Methodology and Study of Their Anti-Influenza Activity. Eur. J. Med. Chem..

[114] Meng L., Su Y., Yang F., Xiao S., Yin Z., Liu J., Zhong J., Zhou D., Yu F. (2019). Design, Synthesis and Biological Evaluation of Amino Acids-Oleanolic Acid Conjugates as Influenza Virus Inhibitors. Bioorg. Med. Chem..

[115] Li W., Yang F., Meng L., Sun J., Su Y., Shao L., Zhou D., Yu F. (2019). Synthesis, Structure Activity Relationship and Anti-Influenza A Virus Evaluation of Oleanolic Acid-Linear Amino Derivatives. Chem. Pharm. Bull..

[116] Shao L., Su Y., Zhang Y., Yang F., Zhang J., Tang T., Yu F. (2023). Nine-Valent Oleanolic Acid Conjugates as Potent Inhibitors Blocking the Entry of Influenza A Virus. Eur. J. Med. Chem..

[117] Purcell R.H. (1994). Hepatitis Viruses: Changing Patterns of Human Disease. Proc. Natl. Acad. Sci. USA.

[118] He W., Gao Y., Wen Y., Ke X., Ou Z., Li Y., He H., Chen Q. (2021). Detection of Virus-Related Sequences Associated with Potential Etiologies of Hepatitis in Liver Tissue Samples from Rats, Mice, Shrews, and Bats. Front. Microbiol..

[119] Yan W., Zhang C., Li B., Xu X., Liang M., Gu S., Chu F., Xu B., Ren J., Wang P. (2016). A Series of Oleanolic Acid Derivatives as Anti-Hepatitis B Virus Agents: Design, Synthesis, and in Vitro and in Vivo Biological Evaluation. Molecules.

[120] Zhang Y., Li J., Zhao J., Wang S., Pan Y., Tanaka K., Kadota S. (2005). Synthesis and Activity of Oleanolic Acid Derivatives, a Novel Class of Inhibitors of Osteoclast Formation. Bioorg. Med. Chem. Lett..

[121] Li J.-F., Zhao Y., Cai M.-M., Li X.-F., Li J.-X. (2009). Synthesis and Evaluation of a Novel Series of Heterocyclic Oleanolic Acid Derivatives with Anti-Osteoclast Formation Activity. Eur. J. Med. Chem..

[122] Wu J., Bao B.-H., Shen Q., Zhang Y.-C., Jiang Q., Li J.-X. (2016). Novel Heterocyclic Ring-Fused Oleanolic Acid Derivatives as Osteoclast Inhibitors for Osteoporosis. MedChemComm.

[123] Zhang Y., Shen Q., Zhu M., Wang J., Du Y., Wu J., Li J. (2020). Modified Quinoxaline-Fused Oleanolic Acid Derivatives as Inhibitors of Osteoclastogenesis and Potential Agent in Anti-Osteoporosis. ChemistrySelect.

